# Structural and functional stability of the sulfur-free surfactant protein B peptide mimic B-YL in synthetic surfactant lipids

**DOI:** 10.1186/s12890-021-01695-0

**Published:** 2021-10-22

**Authors:** Frans J. Walther, Shantanu Sharma, Larry M. Gordon, Alan J. Waring

**Affiliations:** 1 Department of Pediatrics, David Geffen School of Medicine, University of California Los Angeles, 405 Hilgard Avenue, Los Angeles, CA 90095 USA; 2 Lundquist Institute for Biomedical Innovation at Harbor-UCLA Medical Center, 1124 West Carson Street, Torrance, CA 90502 USA; 3 Materials and Process Simulation Center, California Institute of Technology, 1200 East California Boulevard, Pasadena, CA 91125 USA; 4 Department of Medicine, David Geffen School of Medicine, University of California Los Angeles, 405 Hilgard Avenue, Los Angeles, CA 90095 USA

**Keywords:** Synthetic lung surfactant, Surfactant protein B, B-YL peptide, Mass spectroscopy, Fourier-Transform InfraRed Spectroscopy, Homology modeling, Molecular dynamics, Captive bubble surfactometry, Surfactant-deficient rabbits

## Abstract

**Background:**

Optimal functionality of synthetic lung surfactant for treatment of respiratory distress syndrome in preterm infants largely depends on the quality and quantity of the surfactant protein B (SP-B) peptide mimic and the lipid mixture. B-YL peptide is a 41-residue sulfur-free SP-B mimic with its cysteine and methionine residues replaced by tyrosine and leucine, respectively, to enhance its oxidation resistance.

**Aim:**

Testing the structural and functional stability of the B-YL peptide in synthetic surfactant lipids after long-term storage.

**Methods:**

The structural and functional properties of B-YL peptide in surfactant lipids were studied using three production runs of B-YL peptides in synthetic surfactant lipids. Each run was held at 5 °C ambient temperature for three years and analyzed with structural and computational techniques, i.e., MALDI-TOF mass spectrometry, ATR-Fourier Transform Infrared Spectroscopy (ATR-FTIR), secondary homology modeling of a preliminary B-YL structure, and tertiary Molecular Dynamic simulations of B-YL in surfactant lipids, and with functional methods, i.e., captive bubble surfactometry (CBS) and retesting in vivo surface activity in surfactant-deficient young adult rabbits.

**Results:**

MALDI-TOF mass spectrometry showed no degradation of the B-YL peptide as a function of stored time. ATR-FTIR studies demonstrated that the B-YL peptide still assumed stable alpha-helical conformations in synthetic surfactant lipids. These structural findings correlated with excellent in vitro surface activity during both quasi-static and dynamic cycling on CBS after three years of cold storage and in vivo surface activity of the aged formulations with improvements in oxygenation and dynamic lung compliance approaching those of the positive control surfactant Curosurf®.

**Conclusions:**

The structure of the B-YL peptide and the in vitro and in vivo functions of the B-YL surfactant were each maintained after three years of refrigeration storage.

**Supplementary Information:**

The online version contains supplementary material available at 10.1186/s12890-021-01695-0.

## Background

Respiratory problems in very preterm infants are the result of lung immaturity and lung surfactant deficiency. The introduction of intratracheal therapy with animal-derived lung surfactant for this neonatal respiratory distress syndrome has led to a major advance in the viability and morbidity among very preterm infants [1]. The high production costs of porcine (Curosurf®) and bovine (Alveofact®, Infasurf®, Survanta®) surfactant have led to the development of synthetic lung surfactant preparations consisting of mixtures of surfactant protein B and/or C (SP-B and SP-C) peptide mimics in surfactant lipids [2].

Surfactant dispersions derived from extraction of porcine or bovine lung lavage show both degradation of the protein amino acid residues and conformational structure as a function of time [3, 4]. Such changes in primary and secondary structure correlate with loss of in vitro and in vivo surface activity that attenuates the efficacy of the dispersion used for surfactant therapy. Similarly, a synthetic surfactant preparation composed of the SP-B peptide mimic Super Mini-B (SMB) [5] and chemically synthesized synthetic phospholipids underwent chemical changes when stored for an extended period of time [6]. To minimize these changes in the SMB peptide and its secondary conformation and to optimize its surface activity, we developed a sulfur-free peptide B-YL by substituting cysteine residues in SMB with tyrosine and eliminating the need for oxidative formation of the disulfide linkages to stabilize the peptide structure [7]. Replacing the methionine residues in the sequence of SMB with leucine prevented the oxidation of methionine to the more polar methoxide derivative. Here, we investigate the structure, function and stability of this B-YL peptide in surfactant lipids after prolonged refrigerator storage.

## Materials and methods

### Synthesis and purification of B-YL peptide

All organic solvents for sample synthesis and purification were HPLC grade or better. Phospholipids were purchased from Avanti Polar Lipids (Alabaster, AL 35007). Fmoc ^13^C amino acids used for residue specific labeling were supplied by AnaSpec (Fremont, CA 94555). The B-YL peptide was synthesized using standard Fmoc protocols, cleaved and purified as detailed previously [7].

**Fig. 1.**
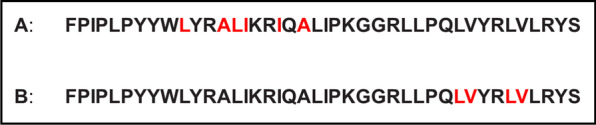
^13^C amino acid labeled residues in red highlight for the N-terminal (**a**) and C-terminal (**b**) predicted helical segments for the 41-residue B-YL peptide for determination of the residue specific secondary structure determination by FTIR

Identification of residue specific amino acid secondary structure conformations were made using ^13^C isotope enhanced versions of the B-YL peptide and are shown in Fig. 1. The B-YL amino acid residues that were designated for ^13^C carbonyl labeling shown in Fig. 1 were based on the predictions from secondary structure homology modeling of the iTasser server (https://zhanglab.ccmb.med.umich.edu/I-TASSER/).

### Formulation of synthetic surfactant dispersions

Peptide and lipids were formulated as lipid-peptide dispersions with a total of 3% by mole fraction of B-YL and 35 mg of total lipid [i.e., DPPC: POPC: POPG 5:3:2 mol:mole:mole] per mL of dispersion [6]. DPPC, POPC and POPG were chosen as lipids because of their similarity with lung composition [8]. The peptide was dissolved in 10 mL of trifluoroethanol and co-solvated with the lipids in chloroform, followed by removal of the solvents with a stream of nitrogen gas and freeze drying of the resulting lipid-peptide film to remove residual solvent. The film was then dispersed with Phosphate Buffered Saline (PBS) and the sample flask containing the hydrated film was rotated for 1 h at 60 °C to produce a solution of multilamellar vesicles (MLVs) [7]. Lipid controls were similarly prepared but without peptide. In the case of ^13^C enhanced amino acid sample sets, the labeled peptide concentration was increased to a mole ratio of peptide to lipid of 1:10 mol:mole to enhance the spectral resolution, so to clearly identify isotope based amide I frequency shifts [9–11]. The dispersions were then stored at 5 °C prior to structural and functional measurements. Since dry samples of the cationic peptide include counter ions, weight per se does not reflect the concentration of the peptide in the synthetic surfactant lipid dispersion, reason why the peptide concentration was quantitated by UV spectroscopy. Direct quantitation of the peptide from aqueous samples was obtained by absorption at 280 nm using the molar extinction coefficient derived from the formalism of [12]. An aliquot of the aqueous formulation was added to HFIP to produce an optically clear sample. The absorption of the lipid component at 280 nm was then corrected by subtraction from the sample using a surfactant lipid sample as a blank. Surfactant lipid concentrations of formulated dispersions were determined by FTIR spectrometry employing the method of Goormaghtigh et al. [13].

### Mass spectral analysis of surfactant B-YL protein and surfactant lipids

Surfactant dispersions containing the B-YL peptide were analyzed using an AB SCIEX TOF/TOF 5800 System (Sciex, Framingham, MA 01701). Samples (~ 50 pmol peptide/µL) were co-solvated with either ∝ -cyano-4-hydroxycinnamic acid or sinapic acid (10 mg matrix/mL water:acetonitrile 1:1, v:v with 0.3% TFA, Sigma Chem Co, Saint Louis, MO 63103) by mixing 24 µL of matrix solution with 1 µL of peptide solution. Two µL of this mixture was then deposited onto a metal Maldi TOF sample plate and allowed to air dry before mass spectral measurement. Instrumental parameters were as follows: mid-mass range, positive linear mode using non-formulated B-YL peptide as an internal standard. The resulting mass spectra were analyzed with AB SCIEX Analyst software and Data Explorer.

### Determination of the secondary conformation of B-YL peptide in synthetic lung surfactant dispersions

ATR-FTIR spectra were recorded at 37 °C using a Bruker Vector 22 FTIR spectrometer (Pike Technologies, Fitchburg, WI 53719) with a deuterium triglyceride sulfate (DTGS) detector. The spectra were averaged over 256 scans at a gain of 4 and a resolution of 2 cm^−1^ [5]. FTIR spectra of B-YL in surfactant lipids, each lipid-peptide solution was transferred onto a germanium ATR crystal. The aqueous solvent was then removed by flowing nitrogen gas over the sample to produce a thick lipid-peptide [5]. The multilayer film was then hydrated to ≥ 35% with deuterated water vapor in nitrogen for 1 h before acquiring the spectra [14]. The spectra for the B-YL peptide in the lipid film was obtained by subtracting the spectrum of a peptide-free control sample from that of the peptide-bound sample.

The relative amounts of α-helix, β-turn, β-sheet, or random (disordered) structures in samples containing non-isotope enhanced B-YL peptides were estimated using Fourier deconvolution (GRAMS/AI 8, version 8.0, Thermo Fisher Scientific, Waltham, MA 02451) and area of component peaks calculated using curve-fitting software (Igor Pro, version 1.6, Wavemetrics, Lake Oswego, OR 97035) [15, 16]. FTIR frequency limits were: α-helix (1662–1645 cm^−1^), β-sheet (1637–1613 cm^−1^ and 1710–1682 cm^−1^), turn/bend (1682–1662 cm^−1^), and disordered or random (1650–1637 cm^−1^) [15, 16].

### Homology modeling of a preliminary structure for B-YL

Preliminary structural models for B-YL were determined by analyzing the respective amino acid sequences with a recent homology modeling program [17, 18]. The homology three-dimensional (3D) structure for B-YL was obtained by first submitting the primary sequence to I-TASSER 5.1 using the automated I-TASSER web service. I-TASSER is a homology algorithm that models discrete regions of the protein using multiple PDB (Protein Data Bank) depositions. The output for a predicted 3D-protein structure was a PDB file, and the accuracy of these models was estimated using such parameters as C-score, TM-score and RMSD [17, 18]. Molecular graphics were rendered using Pymol Version 1.7.4.1 (Schrodinger, LLC; San Diego, CA 92121) or MolBrowser-Pro 3.8-3 (Molsoft LLC; San Diego, CA 92121).

### Secondary structural refinement of B-YL using molecular dynamics

The molecular structure of B-YL was refined with molecular dynamics using the Gromacs suite of programs. The residue specific peptide secondary structure assignments were based on experimental measurements using isotope enhanced FTIR data by placing distance constraints to obtain a final structure (http://www.gromacs.org). The homology structure for B-YL was oriented with respect to the bilayer surface using the Orientations of Proteins in Membranes (OPM) server at http://opm.phar.umich.edu [19]. The system was then minimized using a steepest decent strategy followed by a six-step equilibration process at 311^o^K for a total of 375 ps. This included both NVT (canonical) and NPT (isothermal-isobaric systems) equilibration phases to allow water molecules to reorient around the lipid headgroups and any exposed parts of the peptide as well as permitting lipids to optimize their orientation around the peptide. Equilibration protocols employed a particle-mesh Ewald (PME) strategy for Coulombic long-range interactions and Berendsen temperature coupling. A Berendsen strategy was also used for pressure coupling in a semi-isotropic mode to emulate bilayer motion. After equilibration the system was subjected to a dynamics production run at the same temperature using the Nose–Hoover protocol and pressure (Parrinello-Rahman) values used in the pre-run steps for a period of 500 ns. The Verlet cut-off scheme was employed for all minimization, equilibration, and production steps. Detailed protocols and parameter files for this type of membrane simulation are available from the CHARMM-GUI (http://www.charmm-gui.org) version 2020.3. The output of the production run simulation was analyzed with the Gromacs suite of analysis tools, while molecular graphics were rendered using PyMOL (version 1.7.4.1; Schrödinger, LLC). The final B-YL coordinate set derived from the isotope enhanced FTIR experimental data and refined with molecular dynamics (Gromacs version 2020.3) is available for the lowest energy conformer of the peptide in PDB format (Additional file 1).

### Surface activity using captive bubble surfactometry

Using a fully computerized version of the captive bubble surfactometer described by Schürch and co-workers [5, 7, 20], we measured initial and post-expansion adsorption and quasi-static and dynamic surface tension lowering ability of B-YL surfactant at a physiological cycling rate, area compression, temperature, and humidity [5, 7, 20]. Good surfactants have a minimum surface tension of < 5 mN/m. Here, a minimum surface tension < 2 mN/m during quasi-static cycling and an area compression of maximally 30% to reach a minimum surface tension of < 2 mN/m in the first cycle during dynamic cycling were considered to reflect excellent surface activity. All measurements were performed in triplicate.

### Animal studies

Animal study protocols were reviewed and approved by the Institutional Animal Care and Use Committee of the Lundquist Institute for Biomedical Innovation at Harbor-UCLA Medical Center (# 020645). Procedures and anesthesia followed American Veterinary Medical Association (AMVA) guidelines. Fourteen young adult, male New Zealand white rabbits were purchased from IFPS Inc. (Norco, CA 92860). Surfactant treatment was allocated using dynamic randomization and the sample size of each group was determined by effect size [7]. The study is reported in accordance with ARRIVE guidelines (https://arriveguidelines.org).

In vivo surface activity of aged B-YL surfactant productions was investigated in young adult, male New Zealand white rabbits with a weight of 1.0–1.4 kg who were mechanically ventilated after being made surfactant-deficient with repeated lung lavages of pre-warmed normal saline. Anesthesia, medical care, ventilatory support, and lung function measurement (oxygenation and dynamic lung compliance) have been reported previously [5, 7, 21]. In brief, young adult New Zealand white rabbits (weight 1.0–1.4 kg) were intubated and ventilated with a rodent ventilator and underwent repeated lung lavages with prewarmed normal saline until surfactant deficiency was reached, defined as a partial pressure of oxygen in arterial blood (PaO_2_) < 100 mmHg in 100% oxygen and dynamic compliance < 50% of pre-lavage measure. Surfactant was then administered as an intratracheal bolus at a dose of 100 mg lipids/kg for B-YL surfactant and lipids only (negative control) and at a dose of 200 mg lipids/kg for the porcine surfactant Curosurf® (Chiesi Farmaceutici, Parma, Italy) (positive control). PaO_2_ and dynamic compliance measurements were thereafter followed every 15–30 min for 2 h. Animals were sacrificed 2 h after surfactant administration with an overdose (200 mg/kg) of intravenous pentobarbital.

### Statistical analysis

Data are expressed as mean and standard error of the mean (SEM). Discrete data points were compared with Student’s t-tests. Analysis of functional data was done with one-way analysis of variance (ANOVA) with Tukey’s post-hoc test using SPSS software. Differences between groups were considered statistically significant if the P value was less than 0.05.

## Results

### Mass spectral analysis of surfactant dispersions

**Fig. 2:**
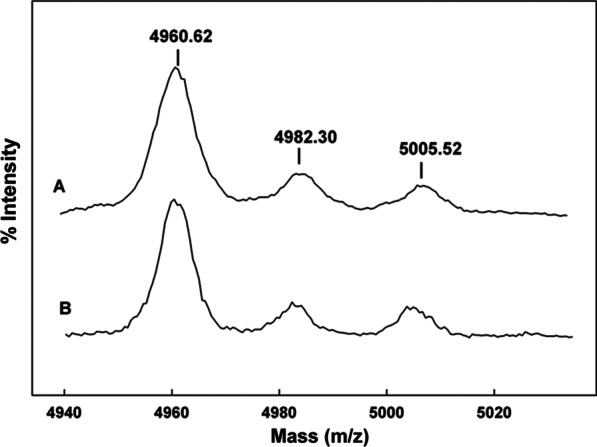
Maldi TOF spectra of B-YL peptide in formulations of synthetic lung surfactant lipids as a function of storage time. B-YL-surfactant lipid samples were prepared for mass spectral analysis as described in Materials and Methods section. **a** Maldi TOF spectrum of a two day old B-YL peptide from a freeze-dried lipid-peptide sample (DPPC:POPC:POPG, 5:3:2 mol:mole). **b** Maldi TOF spectrum of a 3-year old B-YL peptide from a freeze-dried lipid-peptide sample (DPPC:POPC:POPG, 5:3:2 mol:mole)

Mass spectral analysis of the B-YL peptide in surfactant lipid dispersions is shown in Fig. 2. The peptide mass of samples determined a few days after formulation (Fig. 2a) is consistent with that of that predicted for the B-YL peptide. In addition to the spectral peak centered around 4960.62 Da, there were two peaks centered near 4982.30 Da and 5005.52 Da that represent the sodium adducts of the peptide [M + Na]^+^. This is typical of the mass spectra of peptides that absorb alkaline atoms from the solution or glass storage containers and does not represent degradation or chemical modification of the parent peptide [22]. A similar mass spectral pattern was observed in samples stored for three years (Fig. 2b). These findings suggest that there is no degradation of the B-YL peptide formulated in synthetic surfactant lipids and stored under refrigeration at 5 °C for a three-year period.

### Structural conformations of the B-YL peptide in surfactant lipids

**Fig. 3:**
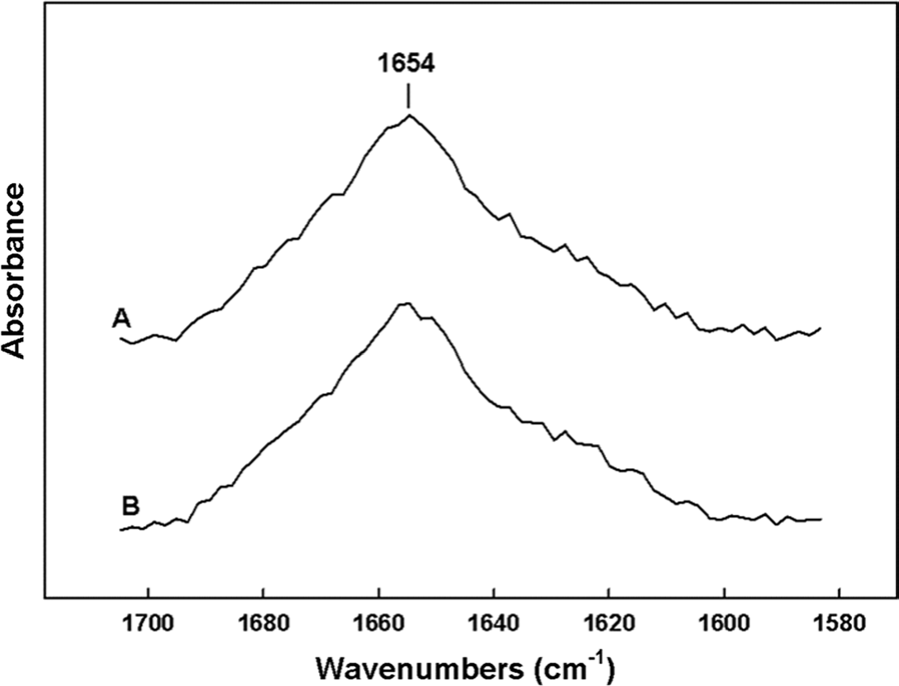
FTIR spectra of B-YL peptide in formulations of synthetic lung surfactant lipids as a function of storage time. B-YL-surfactant lipid samples were prepared for mass spectral analysis as described in Materials and Methods section. **a** FTIR spectrum of a two day old B-YL peptide from a lipid-peptide sample (DPPC:POPC:POPG, 5:3:2 mol:mole) **b** FTIR spectrum of a 3 year old B-YL peptide from lipid-peptide sample (DPPC:POPC:POPG, 5:3:2 mol:mole)

The secondary conformation of the B-YL peptide formulated with synthetic surfactant lipids was also determined using FTIR. The peptide amide I conformational band spectra of the B-YL in synthetic surfactant lipids as a function of time is shown in Fig. 3. Over the three-year period of storage all B-YL peptide in surfactant lipid retained a dominant alpha helical conformation with an absorption centered around 1654 cm^−1^_._ Deconvolution of the amide I band (1700–1600 cm^−1^) into representative conformation contributions is detailed in Table 1. All sample times over the three-year storage period indicate that the B-YL peptide assumed consistent levels for all secondary conformations.

**Table 1:** Proportions of different components of secondary structure for B-YL peptide in surfactant lipid formulations based on FTIR spectroscopic analysis

Sample*	% Conformation			
	α-Helix	Loop-Turn	ß-Sheet	Disordered
B-YL (2 days)	42.5	23.2	13.6	20.4
B-YL (3 years)	41.9	24.5	14.7	18.9

*Peptides in synthetic surfactant dried films hydrated with D_2_O were analyzed for secondary conformation based on secondary structural analysis using spectral deconvolution of the ATR-FTIR spectra of the peptide amide I band (see Methods)

**Fig. 4:**
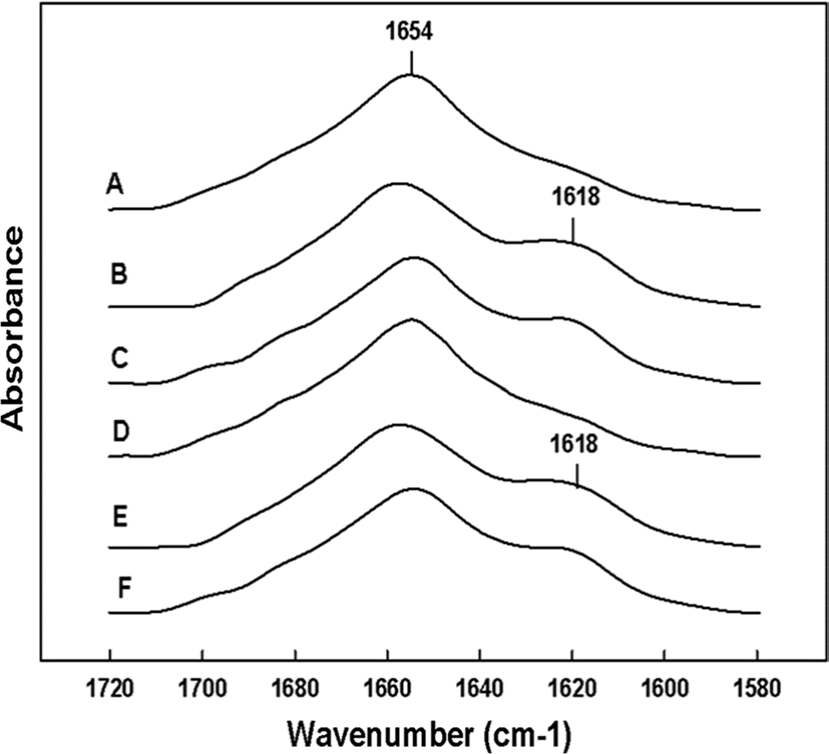
Fourier transform infrared (FTIR) spectra of the amide I band for two-day old preparations **a** ^12^C = O (non-isotopically enhanced) B-YL, **b** ^13^C = O N-terminal helical domain of labeled B-YL (amino acid residues: L_10_/A_13_/L_14_/I_15_/I_18_/A_20_) and **c** ^13^C = O C-terminal helical domain labeled B-YL (amino acid residues: L_32_/V_33_/L_36_/V_37_). Synthetic surfactant samples stored at 5 °C for 3 years **d** ^12^C = O (non-isotopically enhanced) B-YL, **e** ^13^C = O N-terminal helical domain of labeled B-YL (amino acid residues: L_10_/A_13_/L_14_/I_15_/I_18_/A_20_) and **f** ^13^C = O C-terminal helical domain labeled B-YL (amino acid residues: L_32_/V_33_/L_36_/V_37_). Peptides were formulated by co-solvating with surfactant lipids (DPPC:POPC:POPG, 5:3:2 mol:mole) at a ratio of peptide to lipid of 1:10 mol:mole, freeze dried and dispersed into multilamellar vesicles in PBS buffer as detailed in Methods. After storage of the formulated synthetic surfactant for a given period of time, a sample of the dispersion was dried onto the germanium ATR sample plate with a stream of dry nitrogen gas. The dry peptide-lipid film was then hydrated by passing nitrogen gas saturated with D_2_O for one hour before spectral measurements

Although conventional FTIR gives an accurate assessment of the overall secondary conformation of the B-YL peptide in surfactant lipid films, it does not provide residue specific amino acid structural information. To address possible changes in specific residues in the B-YL peptide as a function of time, the B-YL amino acid sequence was enhanced at specific residues with ^13^C carbonyl labeled amino acids that were predicted by homology templating of the B-YL sequence to assume well defined helical conformations (Fig. 1). Comparison of the ^13^C labeled B-YL samples with unlabeled peptide (Fig. 4a) are shown in Figs. 4b–f. Selective isotope enhancement of the N-terminal domain of the B-YL peptide (Fig. 4b) of samples stored for a very short period of time, had a definitive absorbance shift of ~ 36 cm^−1^ from 1654 to 1618 cm^−1^ indicating that residues 10 to 20 assume alpha helical secondary conformations in synthetic surfactant lipids. FTIR spectra of the B-YL C-terminal domain with predicted alpha helical residues 32 and 37 isotopically enhanced (Fig. 4c) also have a ~ 36 cm^−1^ absorbance shift from 1654 to 1618 cm^−1^ also confirming this domain has high helical propensity in synthetic surfactant lipid dispersions. FTIR examination of the above isotope enhanced B-YL—surfactant lipid samples stored at 5 °C for a period of three years also have the same spectral shift associated with the both the N-terminal and C-terminal domains (Fig. 4d–f) as seen with day old samples. These infrared spectral observations indicate that not only does the B-YL peptide have an overall secondary conformational stability when stored for a three-year period, the conformation of peptide on a residue specific basis is unaltered as a function of time.

### Molecular refinement of B-YL structure with molecular dynamics (MD)

Based on experimental isotope enhanced residue specific data from FTIR measurements, MD simulations were subsequently conducted to obtain residue-specific information on B-YL in a surfactant lipid bilayer-water box. Although the above ^13^C-FTIR results on B-YL are useful for experimentally assessing secondary structures averaged over the entire peptide, they cannot indicate the conformations of individual amino acids in the peptide amino acid sequence. With starting models based on homologous structures, however, MD runs in the Gromacs environment provide our most accurate estimates of not only the 3D-conformations of SP-B mimics, but also the molecular topography of these peptides in the hydrated lipid bilayer. In the present work, MD simulations on B-YL were begun by first predicting the homologous 3D-structure by submitting the B-YL amino acid sequence to the I-TASSER web service. Three distinct models for B-YL were obtained from I-TASSER Version 5.1, and Model 1 with the highest C-score was selected. The accuracy of Model 1 was estimated from the following parameters: C-score of -0.26, TM-score of 0.68 ± 0.12 and RMSD of 2.8 ± 2.0 Å. C-score is a confidence score for evaluating the quality of I-TASSER models (between -5 to 2), with elevated values indicating a model with high confidence [17, 18]. TM-score is a scale quantifying the similarity between two structures, with scores > 0.50 signifying a model of correct topology and scores < 0.17 implying random similarity [17, 18, 23, 24]. Moreover, root mean square deviation (RMSD) is an average distance of all residue pairs in the two structures. The high C- and TM-scores, combined with the low RMSD, indicate that Model 1 will provide useful initial estimates of the secondary and tertiary structures for B-YL.

The resulting I-TASSER model for B-YL predicts a C-terminal α-helix (residues P30-V37) connected to an N-terminal helix (residues Y8-L21) via a coil (residues I22-L29), which assumes a helix hairpin conformation [25, 26]. The putative helix hairpin of B-YL executes a reverse turn after 8-residues with the I22-G25 component being the most prominent. Comparable to prior helix hairpins [27], homology analysis of the B-YL sequence indicated high β-turn propensities for I22 to G25, allowing close interactions between the hydrophobic interfaces of the nearly antiparallel N- and C-helices. The helix hairpin fold of B-YL may be stabilized by a general increase in hydrophobicity due to Tyr and Leu substitutions. Enhanced helix hairpin folding may also be due to the formation of clustered Tyr residues (i.e., Y7, Y8, Y11, Y34, and Y40) in the protein interior linking together the N- and C-helices through noncovalent interactions involving aromatic rings. The driving force behind this Tyr networking is at least partly due to “π-stacking” interactions of neighboring aromatic groups [28, 29], which were frequently observed in a survey of proteins deposited in the PDB [30]. Interestingly, Y7, Y8, and Y40 are oriented in a “pinwheel” arrangement with a threefold axis inside the protein core, similarly to how π-stacking interactions optimally organize benzene trimers in an early molecular mechanics study [30]. A second π-stacked configuration for the remaining Tyr residues was identified immediately adjacent to the pinwheel trimer, in which Y34 and Y11 nearly adopt a 1p dimer configuration with their tyrosine rings off-centered and parallel displaced [28, 30]. The I-TASSER model further forecasts a flexible coil for the N-terminal insertion sequence (F1-P6), which permits this hydrophobic segment to interact with the π-stacked tyrosine residues within the B-YL interior.

**Fig. 5:**
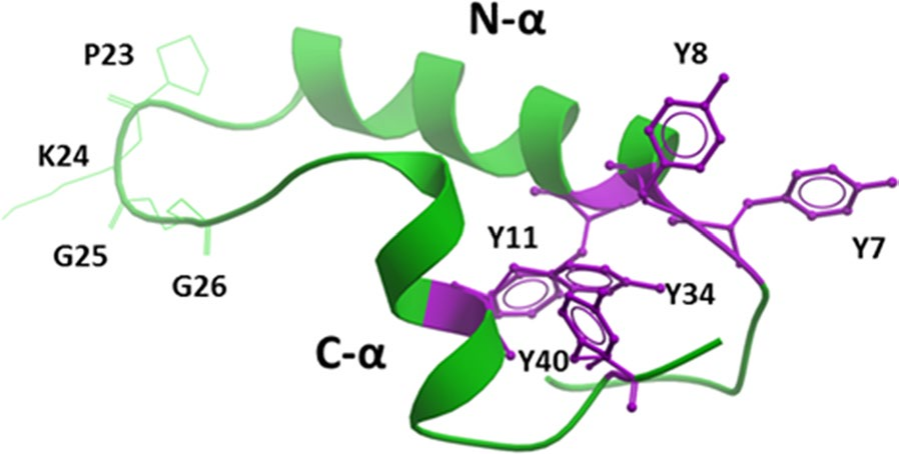
The evolved 3D model for B-YL in lipid bilayers after MD simulations. Molecular Dynamics (MD) simulations were carried out on the B-YL peptide. Main-chain folding of the peptide is displayed in ribbon format, with lipids and water omitted for clarity. Here, the green ribbon MD model for B-YL is shown, in which Tyr replaces Cys at residues 8, 11, 34, and 40 of the parent SP-B peptide mimic Super Mini-B (SMB) with purple stick sidechains (6). This 500 ns simulation predicts a helix-hairpin, in which an N-terminal α-helix (i.e., green N-α, residues R12— L21) connects to a C-terminal α-helix (i.e., green C-α, residues P30—L36) via a turn (green residues I22—L29). The B-YL model also predicts flexible coils for the N-terminal insertion (F1—Y11) and the C-terminal (V37—S41) sequences. The B-YL fold is stabilized by a hydrophobic core of clustered Tyr (Y11, Y34 and Y40) linking together the N- and C-helices through noncovalent interactions involving aromatic rings. The parent (Y7) and substituted (Y8, Y11, Y34 and Y40) tyrosines are shown as purple sticks, and are clustered to the right of the figure

**Fig. 6:**
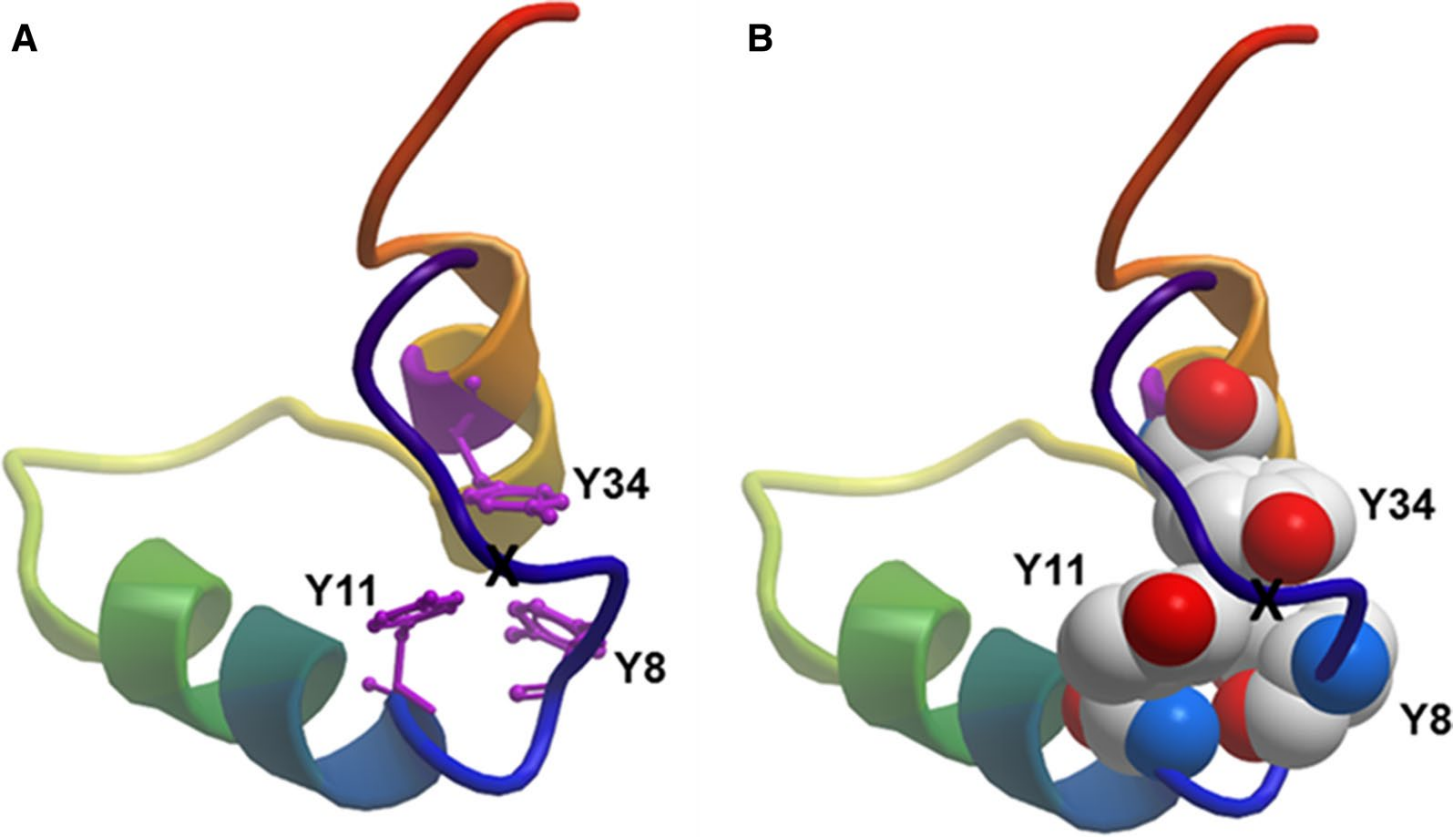
The N- and C-helices of 500 nsec B-YL cross-linked by a symmetry-related tyrosine trimer. Main-chain folding of 500 nsec B-YL is shown in the ribbon-rainbow format, with the N-terminal insertion sequence in blue, the N-terminal helix in blue-green, the turn hairpin in green yellow and the C-terminal helix in yellow-orange. Three of the tyrosine residues (i.e., Y8, Y11, and Y34) are organized as a distorted pinwheel trimer in the hydrophobic interior of the 500 nsec B-YL that links together the N- and C-helices through non-covalent interactions. An approximate threefold axis relating the three tyrosines in Fig. 5a is seen more clearly by rotating the 500 nsec model so that the symmetry axis is perpendicular to the plane of the paper (see “X”). This three-fold axis is designated an “approximate” symmetry axis because it is strictly valid for all three tyrosine ring structures, but not for the hydroxyl of Y8, which faces an opposite direction than those of the hydroxyls for either Y11 or Y34. **a** Graphical representation of the rotated 500 nsec B-YL model is in ribbon-rainbow format, with tyrosine sidechains as purple sticks. **b** Graphical representation of the rotated 500 nsec B-YL model is in ribbon-rainbow format, with tyrosine sidechains as space-filling model

MD simulations of B-YL were next calculated by porting the above I-TASSER Model 1 into aqueous DPPC:POPC:POPG bilayers with potassium counterions to maintain electrical neutrality. This lipid mixture was chosen for in silico studies because it optimizes the surfactant activity of our SP-B mimics in both in vitro and in vivo assays. Simulations were then carried out for 0–500 nsec using the Charmm 36 m implementation for lipids in the Gromacs (Version 2020.3) environment [31]. The representative “500 nsec” structure for B-YL in lipids (Fig. 5) largely agrees with the I-TASSER model on which it is based. Similar to the original I-TASSER model, Fig. 5 indicates that the “500 nsec” model in surfactant lipids is folded as a helix-hairpin-helix, in which an N-terminal α-helix (residues R12-L21) connects to a C-terminal α-helix (P30-L36) via a turn-loop (I22-L29). The high β-turn propensity of B-YL in Fig. 6 (i.e., P23-G26; green wire sidechains) allows close interaction between the hydrophobic interfaces of the nearly antiparallel N- and C-helices. The 500 ns B-YL model also predicts flexible coils for the N-terminal insertion (F1-Y11) and the C-terminal (V37-S41) sequences. Lastly, space-filling I-TASSER and 500 ns MD simulation models for B-YL each produced a compact globular protein, exhibiting a core of hydrophobic residues and a surface of aqueous-exposed and polar residues.

Although major changes were not observed between the I-TASSER and 500-nsec models for B-YL, there were nevertheless significant minor differences which we attribute to the 500 nsec-equilibration of the I-TASSER structure in the aqueous lipid-bilayer box. For example, note that the N α-helix of the “500 nsec” model is shorter by four residues, with Y8, W9, L10, and Y11 not participating in the N α-helix. Concurrently with the fraying of the N-helix by one turn, there is a reorganization of the five tyrosine residues in the “500 nsec” model. Namely, the pinwheel trimer and 1p dimer in the I-TASSER B-YL model convert to a distorted pinwheel trimer (i.e., Y8, Y11 and Y34) in the hydrophobic interior of the 500 nsec B-YL, with Y7 and Y40 migrating to the polar surface (Fig. 6). The approximate threefold axis relating the three tyrosines is seen more clearly by reorienting the 500 nsec model so that the symmetry axis is perpendicular to the plane of the paper (see “X"), with the Tyr representations in purple stick (Fig. 6a) and space-filling (Fig. 6b), respectively. This three-fold axis is designated an “approximate” symmetry axis because it is strictly valid for all three tyrosine ring structures, but not for the hydroxyl of Y8, which faces an opposite direction than those of the hydroxyls for either Y11 or Y34 (Fig. 6a, b). Figure 6 confirms that the 500 nsec model B-YL folds as a helix hairpin, with the approximate pinwheel trimer (Y8, Y11, and Y34) bridging together the N- and C-α helices through non-covalent interactions. The space-filling 500 nsec model for B-YL further demonstrates a globular protein, in which the pinwheel Tyr trimer embeds with other hydrophobic residues in the interior, while hydrophilic groups face the exterior (*not shown*). Notably, the pinwheel Tyr trimer in Fig. 6 may act as a hydrophobic core around which other hydrophobic residues assemble, but from which all water is absent. Because water disrupts secondary structure by attacking amide-amide hydrogen bonds, the stability of the B-YL conformation for 500 nsec in Figs. 6a, b may be due not only to non-covalent interactions between hydrophobic residues (e.g., π-stacking of Tyr residues) but also to water exclusion from the hydrophobic interior (i.e., solvophobic forces) [28, 29].

**Fig. 7:**
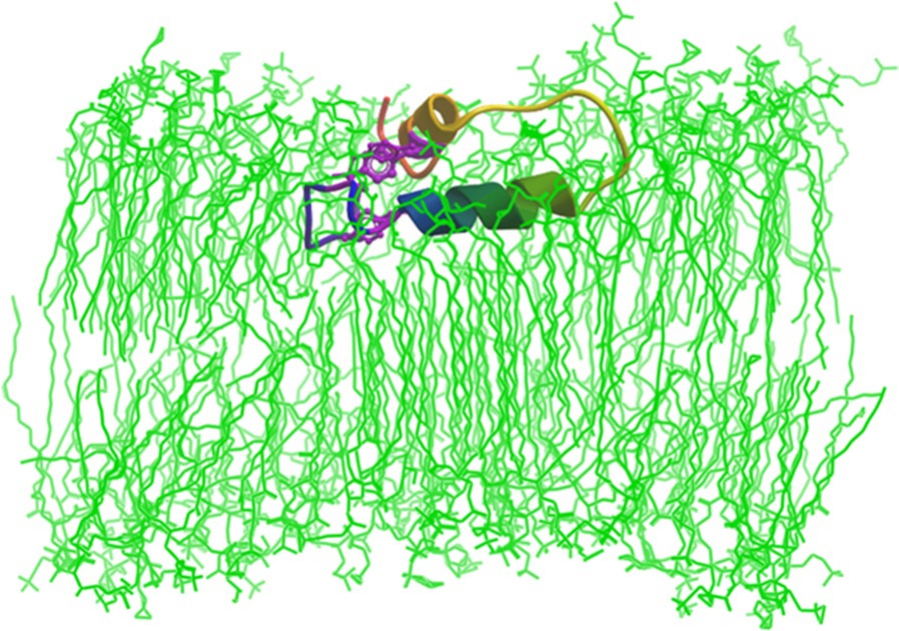
Cross-sectional views of the B-YL peptide in lipid surfactant bilayers after MD simulations. Main-chain folding of the B-YL peptide is shown in the ribbon-rainbow format, with the N-terminal insertion sequence in blue, the N-terminal helix in blue-green, the turn hairpin region in green-yellow and the C-terminal helix in yellow-orange. Lipids are shown as green stick figures, while water is left out for clarity. The ribbon model for the 500 nsec B-YL conformer folds as a helix-hairpin, with the Y8, Y11 and Y34 tyrosine residues (i.e., purple stick figures) bridging the N- and C-α helices through noncovalent aromatic interactions. The axes for the N- and C-α helices are each nearly parallel to the bilayer plane. However, the N-α helix lies deeper in the bilayer subjacent to the lipid headgroup, while the C-α helix binds to the more polar lipid-water interface

The topological organization of B-YL in the membrane bilayer may also be characterized using the above all-atom (500 nsec) MD simulation of B-YL in hydrated lipid surfactant (DPPC:POPC:POPG 5:3:2 mol:mole:mole) at 37 °C. Figure 7 shows a cross-sectional view of the ribbon model for the 500 nsec B-YL in the lipid bilayer. Here, the buried Y8, Y11 and Y34 tyrosines (i.e., purple stick sidechains) bridge the N- and C-α helices through noncovalent aromatic interactions, while the surface-exposed Y7 and Y40 interact with water molecules or polar lipid headgroups. The axes for the N- and C-α helices are each nearly parallel to the bilayer plane. However, the N-α helix lies deeper in the bilayer subjacent to the lipid headgroup, while the C-α helix binds to the more polar lipid-water interface (Fig. 7). This differential partitioning may be just due to differences in the hydrophobicities of the two helices because MPEx analysis [32] indicated a higher hydropathy (i.e., more hydrophobic) for the N-α-helix than for the C-α helix (i.e., 4.92 *vs.* −0.02 kcal/mol, respectively). The 500 nsec—MD simulation model for B-YL in aqueous surfactant lipids also demonstrated the absence of any water or lipid within its hydrophobic core (*not shown*). The systematic exclusion of water from the protein interior mediated by the three “π-stacked” tyrosine residues may account for why B-YL folds as a helix-hairpin for simulation times (t) ≤ 500 nsec.

**Fig. 8:**
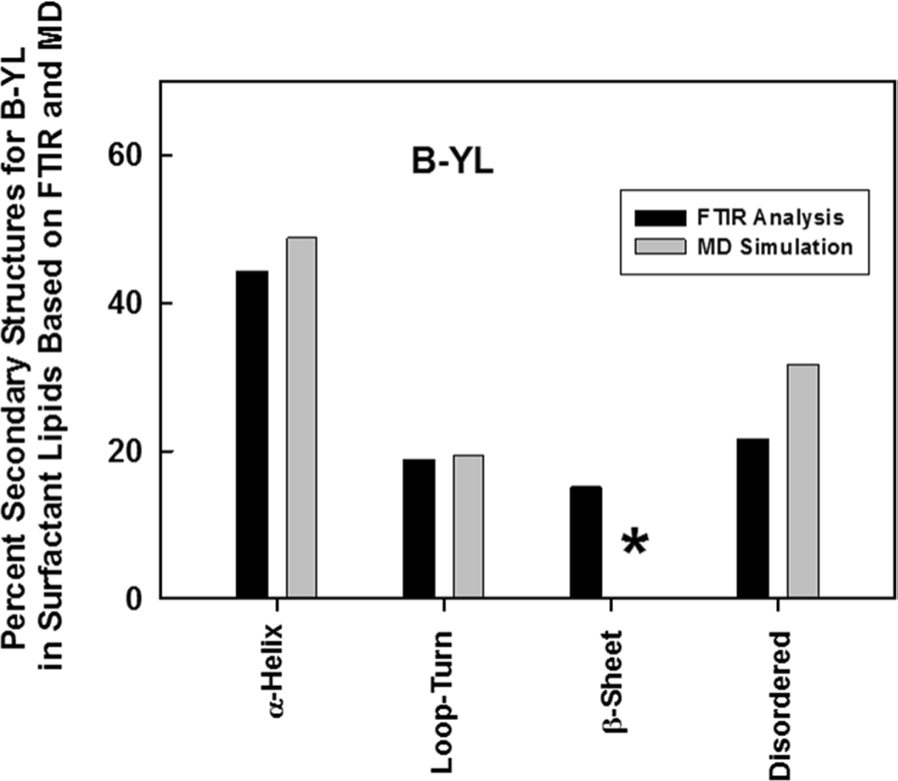
Spectroscopic and MD simulation of the secondary structure for B-YL in lipids. The B-YL peptide lacks disulfide linkages with Tyr substituted for Cys at residues 8, 11, 34 and 40 and also Leu replacing Met at residues 21 and 28 (Fig. 1). Plot of % conformations assessed from FTIR spectra (*black bars*) of the B-YL mimic in DPPC:POPC: POPG (mole:mole:mole, 5:3:2) bilayers (Table 1) *vs.* the corresponding structures calculated from DSSP analysis (i.e., H-bond estimation algorithm) of an MD simulation of B-YL (*gray bars*) in an aqueous DPPC:POPC:POPG lipid box for 500 nsec. The respective proportions of secondary structures for B-YL obtained from FTIR deconvolution are in good agreement with those determined using MD simulations, with each technique showing α-helix > Loop-Turn ~ Disordered ≥ β-sheet. The absence of any β-sheet in our MD model is shown by the asterisk (*)

Importantly, partial validation of the 500 nsec MD structure for B-YL in aqueous surfactant lipids (Fig. 8) is provided by our above spectroscopic findings. The secondary structures obtained from FTIR spectra of B-YL in surfactant lipids are generally in good agreement with those predicted in the 500 nsec model (Fig. 8). For example, Fig. 8 indicates that the respective proportions of secondary structures for B-YL obtained from FTIR self-deconvolutions are compatible with those determined using MD simulations, with the experimental and theoretical techniques each showing high α-helix and smaller contributions due to loop-turn, disordered and β-sheet. These results indicate experimental confirmation of critical features for our MD model of B-YL folding as an α-helix-hairpin (Fig. 6a).

### Captive bubble surfactometry

**Fig. 9:**
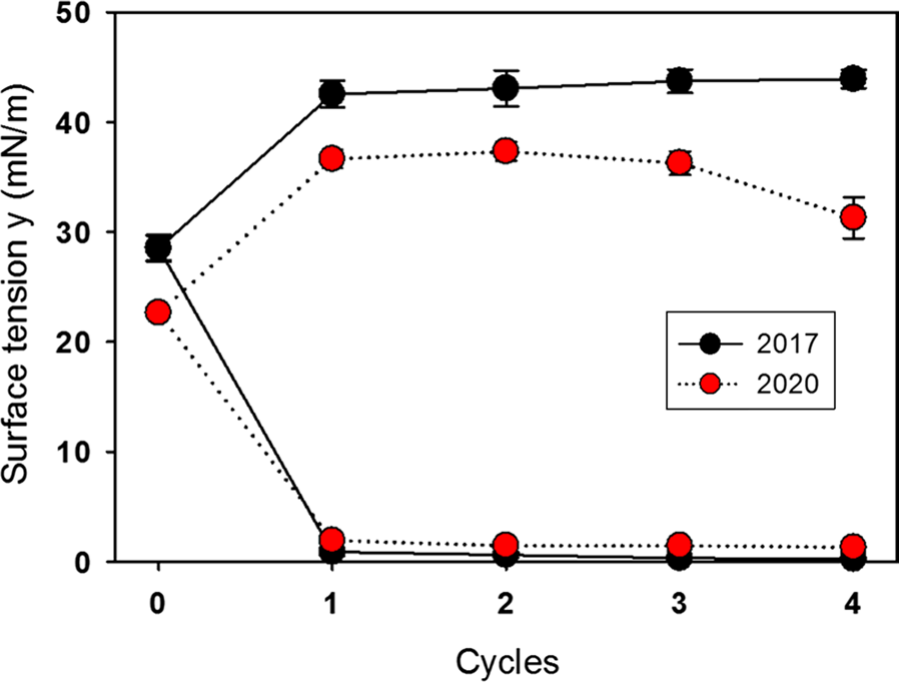
Average ± SEM surface tension values of quasi-static cycles at captive bubble surfactometry of three B-YL surfactant productions produced in 2017 and re-measured in 2020. Minimum surface tension (lower part of the curves) during quasi-static cycling was < 2 mN/m when tested shortly after production and after three years of refrigerated storage. Maximum surface tension (upper part of the curves) decreased slightly over time

**Fig. 10:**
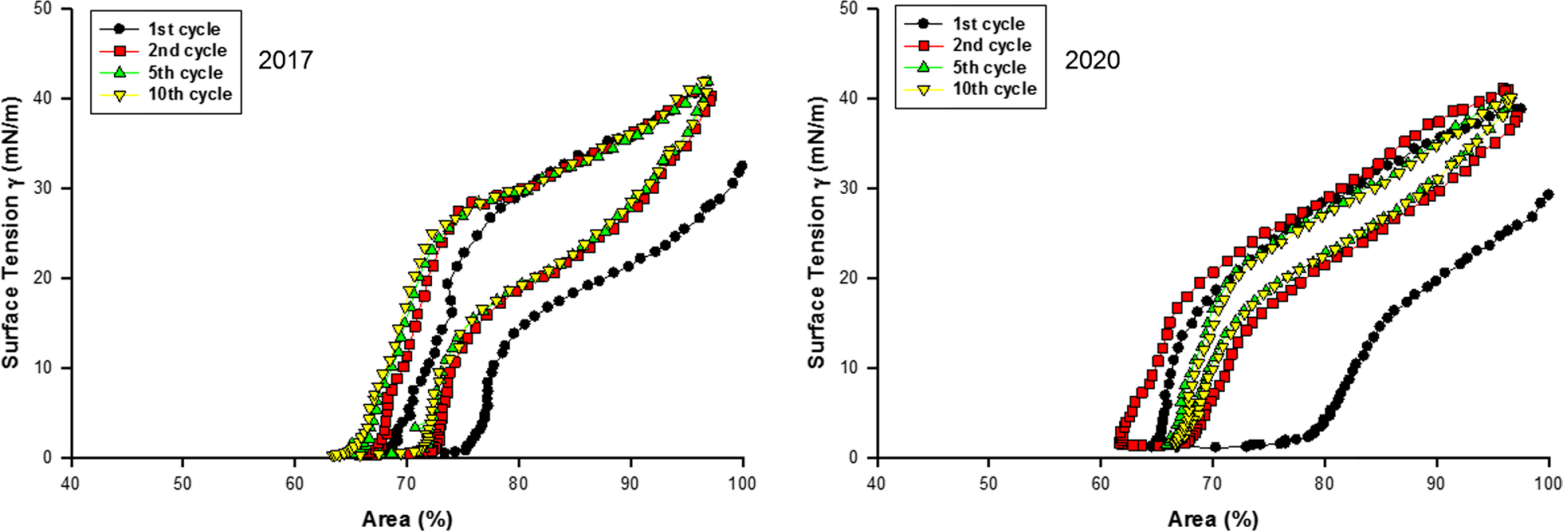
Average surface tension values of dynamic cycles (20/min) at captive bubble surfactometry of 3 B-YL surfactant preparations produced in 2017 and re-measured in 2020. Area reduction to reach a minimum surface tension < 2 mN/m and hysteresis were essentially unchanged after 3 years of refrigerated storage

Surface tension of three B-YL surfactant productions that had originally been measured with captive bubble surfactometry in 2017 were re-measured in 2020 after refrigerator storage for three years. Figure 9 shows their average ± SEM quasi-static cycling data and Fig. 10 their average ± SEM dynamic cycling data. Quasi-static findings did not change over time and showed excellent surface activity with minimum surface tension values < 2 mN/m (Fig. 9). Maximum surface tension tended to decrease slightly over time. Dynamic cycling demonstrated minimum surface tension values < 2 mN/m starting at 20–30% area compression with only a limited decrease in hysteresis surface over time (Fig. 10).

### B-YL surfactant treatment of surfactant-deficient rabbits

**Fig. 11:**
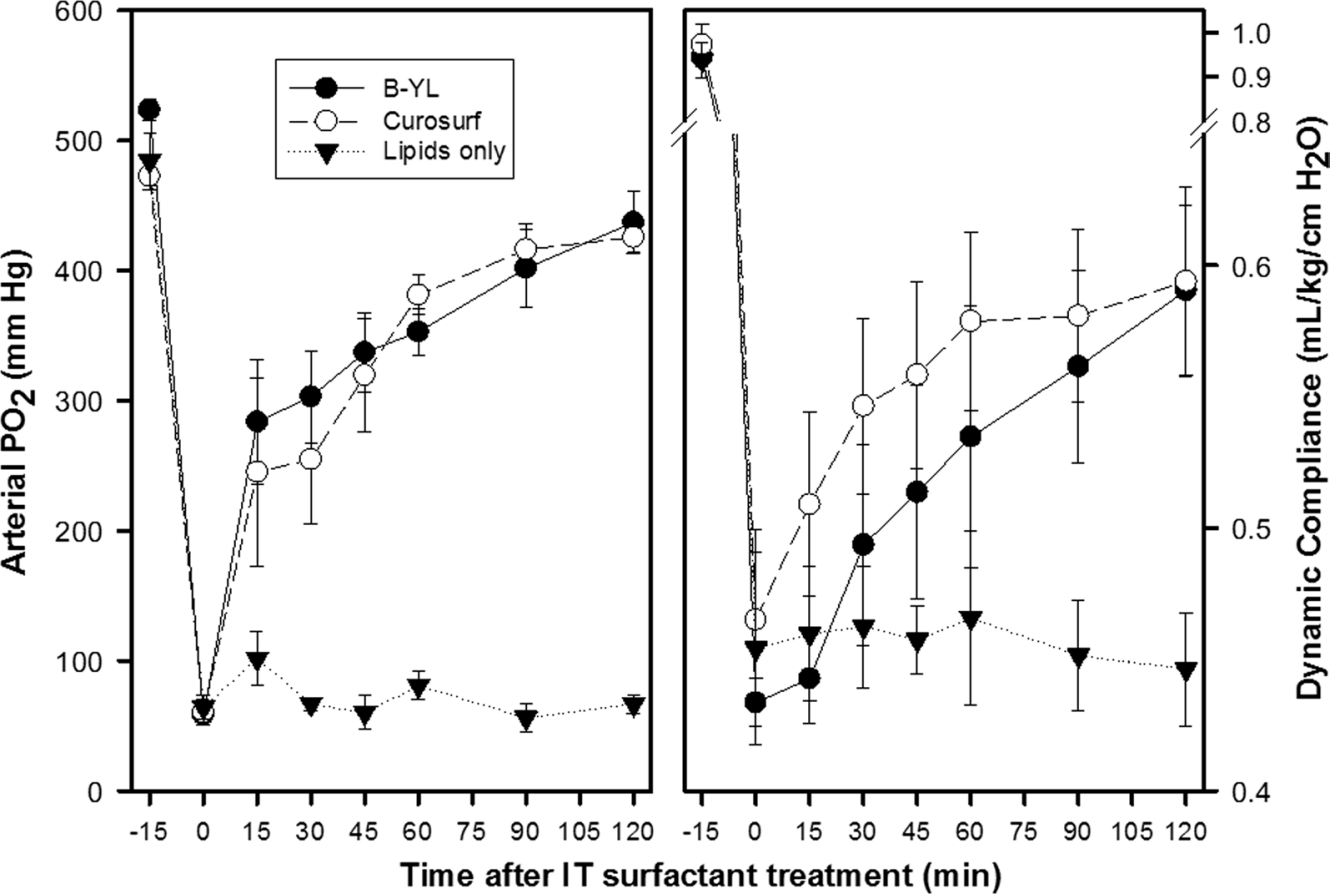
Oxygenation (PaO_2_) and dynamic lung compliance in surfactant-deficient rabbits treated with aged B-YL surfactant (n = 6) compared to porcine surfactant (Curosurf®, n = 4) and lipids only surfactant (n = 4). Surfactant treatment was given intratracheally at time 0 min, after surfactant deficiency was induced by repeated lung lavages with warmed normal saline. Oxygenation (arterial PO_2_ in left-sided graph) quickly increased to levels close to the baseline values and reached similar terminal values after treatment with B-YL and Curosurf®, but did not change significantly after treatment with lipids only. Dynamic compliance followed a similar pattern, but the increase in compliance occurred slower and terminal values for B-YL and Curosurf® were equal but below the baseline levels. Baseline (2017) in vivo data can be found in reference 7

The three B-YL surfactant productions from 2017 were mixed and used for intratracheal administration to surfactant-deficient rabbits. Improvements in oxygenation and dynamic lung compliance during the next 2 h were compared to the porcine surfactant Curosurf® (Chiesi Farmaceutici, Parma, Italy) as positive control and lipids only as negative control. Average post-lavage PaO_2_ values were similar among the three rabbit groups. Oxygenation improved sharply and similarly after treatment with B-YL surfactant and Curosurf®, whereas treatment with lipids only did not affect oxygenation significantly (Fig. 11). Average post-lavage dynamic compliance values were lowest in the B-YL surfactant group. Dynamic compliance increased (as expected) slower in the B-YL and Curosurf® groups than oxygenation. The B-YL group caught up from its lower post-lavage compliance and terminal lung compliance in the B-YL and Curosurf® groups was similar and averaged ~ 25% over post-lavage values. Treatment with lipids only did not significantly affect dynamic lung compliance.

## Discussion

There are a number of changes in the structure and function of lung surfactant components as a function of time that include changes in the hydrophobic lung surfactant proteins and lipids. Some of the changes in protein chemical structure observed are the oxidation of specific amino acid residues. Typical oxidative chemical modifications of native SP-B protein in Bovine lipid extract induced by hypochlorous acid is that of the methionine-29 and 65 to the more polar methoxide derivative [33]. Another residue that has been shown to be susceptible to oxygen mediated damage by Fenton reagent is tryptophan-9 that is transformed to hydroxy-tryptophan, N-formyl-kynurenine and kynurenine [34]. These chemically mediated alterations in the SP-B amino acid result in diminished surface activity of the surfactant dispersion. Clinical surfactant dispersions that contain native SP-B protein also show aromatic amino acid oxidation from exposure of clinical surfactant dispersions to environmental tobacco smoke [33]. Both tryptophan and tyrosine are degraded and all methionine residues were converted to the methoxide derivative. These amino acid changes were accompanied by secondary structure alterations with a loss of the dominant alpha helical conformation to enhanced levels of disordered structures and beta sheet conformations. The changes in SP-B primary and secondary structure correlated with decreased surface activity changes in the lipid film morphology.

It is notable that there is some beta sheet associated with B-YL observed in the samples analyzed by FTIR methodology that is not present in the in silico molecular model of the peptide in synthetic surfactant lipid bilayers. The difference in peptide structure is most likely the result of the limitations of the computer cluster employed in this study. Our present system size only permits the simulation of a single peptide in the surfactant lipid bilayer rather than a large lipid-peptide ensemble that has multiple B-YL peptides that have some self-association properties between peptides to form intramolecular beta sheets.

Changes in the structure of lung surfactant from native derived formulations were also observed in surfactant dispersions made from SP-B peptide mimics and synthetic surfactant lipids. The interaction of ozone with peptide B_1-25_ that spans the N-terminal 25 residues of the SP-B protein human sequence resulted in the heterogeneous oxidation of the peptide at the air–liquid interface of a model surfactant system with DOPG lipids [35]. Interfacial oxidation of the B_1-25_ peptide included methoxide formation derived from methionine 21 and the addition of two oxygens to tryptophan 9 to form N-formyl-kynurenine (NFKyn) over a thirty-second time period. In contrast to interface-based ozonolysis, solution oxidation of SP-B_1-25_ in methanol by bubbling ozone in the peptide-solvent solution for 1 min produced changes in Cys-8 and Trp-9 to sulfonic acid and hydroxy-N-formylkynurenine (HNFKyn), respectivety. The use of Fenton reagent as an oxidant also resulted in formation of NFKyn, HNFKyn and methoxide within five minutes after addition of the reagent.

Long term refrigerated storage at 5 °C of SMB, a highly active disulfide linked N-terminal-C-terminal helix-hairpin mimic of the native SP-B protein in synthetic surfactant lipids aqueous dispersions, also showed some specific oxidation of amino acid residues [6]. There was 17.5% methoxide formation over a five-year period in the peptide-lipid dispersion that was used for in vitro and in vivo testing. However, FTIR measurements of the dispersion films indicated that there was no change in the secondary structure of the peptide with time. Similarly, there was no attenuation of the SMB in vitro and in vivo surface activity as a function of time.

In the present study, the B-YL peptide proved to be very stable with regard to primary sequence amino acid damages. Since the helix hairpin had all cysteines replaced with tyrosine, there was no oxidation of these residues to sulfonic acid. The replacement of methionine amino acids in the sequence with leucine also prevented the oxidation of these residues. These mutations rendered the B-YL peptide more stable to oxidative damage than the SMB parent peptide as a function of storage time over a period of three years. The B-YL peptide also had a very stable overall secondary structure that correlated well with in vitro and in vivo surface activity. Not only did the overall secondary conformation go unchanged with prolonged storage, the residue specific conformation of the alpha helical structure remained the same.

Surfactometry of B-YL surfactant demonstrated that mimimum surface tension during quasi-static and dynamic cycling had not changed over three years in the three surfactant productions investigated. Maximum surface tension values decreased with a slight change in hysteresis over time. These in vitro surface activity data were confirmed in ventilated young adult rabbits. Treatment with B-YL surfactant after induction of surfactant-deficiency by repeated lung lavages led to similar improvements in oxygenation and dynamic lung compliance as treatment with the positive control Curosurf®, whereas treatment with lipids only as negative control did not lead to any improvement.

## Conclusion

Our data indicate that structure and function of mixtures of the SP-B peptide mimic B-YL and surfactant lipids are remarkably stable over a period of three years of refrigerated storage due to their improved resistance against oxidation.

## Supplementary Information

**Additional file 1:** Protein Data Bank atom coordinate Format File for the lowest energy conformer of B-YL peptide dynamics simulation in synthetic surfactant bilayer.

## Data Availability

All data generated or analyzed during this study are included in this published article and additional file 1.

## Abbreviations

ATR-FTIR: Attenuated total reflection Fourier-transform infrared spectroscopy
CBS: Captive bubble surfactometry
DPPC: Dipalmitoylphosphatidylcholine
FTIR: Fourier-transform infrared spectroscopy
HNFKyn: Hydroxy-N-formylkynurenine
MALDI-TOF: Matrix-assisted laser desorption ionization time-of-flight mass spectrometry
MD: Molecular dynamics
NFKyn: N-formyl-kynurenine
PaO_2_: Partial pressure of oxygen
PDB: Protein data bank
POPC: Palmitoyl-oleoyl-phosphatidylcholine
POPG: Palmitoyl-oleoyl-phosphatidylglycerol
SEM: Standard error of the mean
SMB: Super Mini-B
SP-B: Surfactant protein B
