# Supplementary material for: Structural and functional stability of the sulfur-free surfactant protein B peptide mimic B-YL in synthetic surfactant lipids
Source: BMC Pulm Med. 2021 Oct 22;21:330. doi: 10.1186/s12890-021-01695-0 (PMC8540162; doi:10.1186/s12890-021-01695-0)
Supplement: Supplementary file 1 — Additional file 1: Protein Data Bank atom coordinate Format File for the lowest energy conformer of B-YL peptide dynamics simulation in synthetic surfactant bilayer. [file 12890_2021_1695_MOESM1_ESM.docx]

**Additional File 1 for:**

**Structural and Functional Stability of the Sulfur-Free Surfactant Protein B Peptide Mimic B-YL in Synthetic Surfactant Lipids**

Frans J. Walther, M.D., Ph.D.^1,2^

Shantanu Sharma, Ph.D.^3^

Larry M. Gordon, Ph.D.^2^

Alan J. Waring, Ph.D.^2,4^

^1^ Department of Pediatrics, David Geffen School of Medicine, University of California Los Angeles, 405 Hilgard Avenue, Los Angeles, CA 90095

^2^ Lundquist Institute for Biomedical Innovation at Harbor-UCLA Medical Center, 1124 West Carson Street, Torrance, CA 90502

^3^ Materials and Process Simulation Center, California Institute of Technology, 1200 East California Boulevard, Pasadena, CA 91125

^4^ Department of Medicine, David Geffen School of Medicine, University of California Los Angeles, 405 Hilgard Avenue, Los Angeles, CA 90095

**Corresponding author**: Frans J. Walther ([fjwalther@ucla.edu](mailto:fjwalther@ucla.edu))

**Protein Data Bank atom coordinate Format File for the lowest energy conformer of B-YL peptide molecular dynamics simulation in synthetic surfactant lipid bilayer** (**DPPC:POPC:POPG, 5:3:2 mole:mole).**

**HEADER SURFACE ACTIVE PROTEIN 3-MAR-21 B-YL**

**TITLE STRUCTURAL AND FUNCTIONAL STABILITY OF THE SULFUR-FREE SURFACTANT PROTEIN B**

**TITLE 2 MIMIC B-YL IN SYNTHETIC SURFACTANT LIPIDS**

**COMPND MOL_ID: 1;**

**COMPND 2 MOLECULE: PULMONARY SURFACTANT-ASSOCIATED PROTEIN B;**

**COMPND 3 CHAIN: A;**

**COMPND 4 FRAGMENT: RESIDUES 201-218, 263-278;**

**COMPND 5 SYNONYM: SP-B, 6 KDA PROTEIN, PULMONARY SURFACTANT-ASSOCIATED**

**COMPND 6 PROTEOLIPID SPLPHE, 18 KDA PULMONARY-SURFACTANT PROTEIN;**

**COMPND 7 ENGINEERED: YES**

**SOURCE MOL_ID: 1;**

**SOURCE 2 SYNTHETIC: YES;**

**SOURCE 3 ORGANISM_SCIENTIFIC: HOMO SAPIENS;**

**SOURCE 4 ORGANISM_COMMON: HUMAN;**

**SOURCE 5 ORGANISM_TAXID: 9606**

**KEYWDS LUNG SURFACTANT PROTEIN, SAPOSIN, SURFACE ACTIVE PROTEIN**

**EXPDTA INFRARED SPECTROSCOPY**

**NUMMDL 1**

**AUTHOR F.J.WALTHER,S.Sharma,L.M.GORDON,A.J.WARING**

**JRNL AUTH F.J.WALTHER,S.Sharma,L.M.GORDON,A.J.WARING**

**JRNL TITLE STRUCTURAL AND FUNCTIONAL STABILITY OF THE SULFUR-FREE SURFACTANT PROTEIN B**

**JRNL TITLE 2 MIMIC B-YL IN SYNTHETIC SURFACTANT LIPIDS**

**JRNL REF GATES OPEN RESEARCH V. NA ___ 2021 In preparation**

**JRNL REFN NA ISSN NA**

**JRNL PMID NA**

**JRNL DOI NA**

**REMARK 2**

**REMARK 2 RESOLUTION. NOT APPLICABLE.**

**REMARK 4**

**REMARK 4 B-YL COMPLIES WITH FORMAT V. 3.15, 01-DEC-08**

**REMARK 100**

**REMARK 100 THIS ENTRY HAS BEEN PROCESSED BY RCSB ON 3-MAR-21.**

**REMARK 100 THE DEPOSITION ID IS to be issued.**

**REMARK 250**

**REMARK 250 EXPERIMENTAL DETAILS. EXPERIMENT TYPE : INFRARED SPECTROSCOPY.**

**REMARK 250 TEMPERATURE : 311 PRESSURE : 1.0 AMBIENT SAMPLE CONTENTS : THIS**

**REMARK 250 STRUCTURE WAS DETERMINED USING 13-C ISOTOPE ENHANCED FTIR**

**REMARK 250 SPECTROSCOPY ON A FAMILY OF SELECTIVELY LABELED CHEMICALLY**

**REMARK 250 SYNTHESIZED PEPTIDES. 13-C CARBONYL LABELS INCLUDED RESIDUES 10,13**

**REMARK 250 14,15,18,20,32,33,36,37.. PEPTIDE IN SURFACTANT PHOSPHOLIPID MULTILAYER**

**REMARK 250 SOLVATED WITH PBS-D20 VAPOR FTIR EXPERIMENTS CONDUCTED :**

**REMARK 250 DISTANCE GEOMETRY; SIMULATED ANNEALING SPECTROMETER MODEL :**

**REMARK 250 VECTOR 22 FTIR SPECTROMETER MANUFACTURER : BRUKER STRUCTURE**

**REMARK 250 DETERMINATION. SOFTWARE USED : ITASSER 5.1, GOMACS 2021.3, CHARMM 36m**

**REMARK 250 FORECE FIELD**

**REMARK 250 METHOD USED : DISTANCE GEOMETRY; SIMULATED ANNEALING CONFORMERS,**

**REMARK 250 NUMBER CALCULATED : 20 CONFORMERS, NUMBER SUBMITTED : 1**

**REMARK 250 CONFORMERS, SELECTION CRITERIA : STRUCTURES WITH ACCEPTABLE**

**REMARK 250 COVALENT GEOMETRY BEST REPRESENTATIVE CONFORMER IN THIS ENSEMBLE**

**REMARK 250 : 1 REMARK: THE COORDINATES IN THIS ENTRY WERE GENERATED FROM 13-**

**REMARK 250 C INDUCED SPECTRAL SHIFTS WHICH GIVE RESIDUE-SPECIFIC SECONDARY**

**REMARK 250 STRUCTURE INFORMATION. STRUCTURE**

**REMARK 300**

**REMARK 300 BIOMOLECULE: 1**

**REMARK 300 SEE REMARK 350 FOR THE AUTHOR PROVIDED AND/OR PROGRAM**

**REMARK 300 GENERATED ASSEMBLY INFORMATION FOR THE STRUCTURE IN**

**REMARK 300 THIS ENTRY. THE REMARK MAY ALSO PROVIDE INFORMATION ON**

**REMARK 300 BURIED SURFACE AREA.**

**REMARK 350**

**REMARK 350**

**REMARK 350 BIOMOLECULE: 1**

**REMARK 350 AUTHOR DETERMINED BIOLOGICAL UNIT: MONOMERIC**

**REMARK 500**

**REMARK 500 GEOMETRY AND STEREOCHEMISTRY**

**REMARK 500 MODEL QUALITY EVALUATED WITH PROCHECK**

**REMARK 500 http://www.ebi.ac.uk/thornton-srv/databases/pdbsum**

**REMARK 500 RAMACHANDRAN PLOT STATISTICS**

**REMARK 500 RESIDUES iN MOST FAVORDED REGIONS [A,B,L] 87.5%**

**REMARK 500 RESIDUES IN ADDITIONAL ALLOWED REGIONS [a,b,l,p 12.5%**

**REMARK 500 RESIDUES IN GENEROUSLY ALLOWED REGIONS [~a,~b,~l,~p] 0.0%**

**REMARK 500 RESIDUES IN DISALLOWED REGIONS [XX] 0.0%**

**REMARK 500**

**REMARK 500 BETA TURN TABLE**

**REMARK 500**

**REMARK 500 No. Turn Sequence* type Phi Psi Chi1 Phi Psi Chi1 CA-dist H-bond**

**REMARK 500 1. ILE22-GLY25 IPKG VIII -75.8 -41.8 4.8 -137.6 143.0 -64.8 6.7 Yes**

**REMARK 500 2. LEU29-LEU32 LPQL I -67.1 -27.3 0.7 -96.5 -2.4 64.4 5.5**

**REMARK 500 REMARK: NULL**

**REMARK 500 NA**

**REMARK 500**

**REMARK 500 REMARK: NULL**

**DBREF B-YL A 1 41 UNP P07988 PSPB_HUMAN 208 278**

**SEQRES 1 A 41 PHE PRO ILE PRO LEU PRO TYR TRP LEU TYR ARG ALA LEU ILE LYS ARG ILE**

**SEQRES 2 A 41 GLN ALA LEU ILE PRO LYS GLY GLY ARG LEU LEU PRO GLN**

**SEQRES 3 A 41 LEU VAL TYR ARG LEU VAL LEU ARG TYR SER**

**HELIX 1 1 TYR A 8 LEU A 21 1 14**

**HELIX 2 2 LEU A 32 VAL A 37 1 6**

**CRYST1 1.000 1.000 1.000 90.00 90.00 90.00 P 1 1**

**MODEL 1**

**ATOM 1 N PHE A 1 47.190 18.270 43.090 1.00 0.13 N**

**ATOM 2 CA PHE A 1 46.970 18.500 44.530 1.00 0.13 C**

**ATOM 3 C PHE A 1 45.520 18.780 44.840 1.00 0.13 C**

**ATOM 4 O PHE A 1 45.040 19.830 44.490 1.00 0.13 O**

**ATOM 5 CB PHE A 1 47.860 19.700 44.940 1.00 0.13 C**

**ATOM 6 CG PHE A 1 48.350 19.620 46.300 1.00 0.13 C**

**ATOM 7 CD1 PHE A 1 47.830 20.150 47.080 1.00 0.13 C**

**ATOM 8 CD2 PHE A 1 49.260 18.990 46.830 1.00 0.13 C**

**ATOM 9 CE1 PHE A 1 48.220 20.050 48.320 1.00 0.13 C**

**ATOM 10 CE2 PHE A 1 49.680 18.890 48.060 1.00 0.13 C**

**ATOM 11 CZ PHE A 1 49.180 19.430 48.800 1.00 0.13 C**

**ATOM 23 N PRO A 2 44.790 17.900 45.470 1.00 0.17 N**

**ATOM 24 CA PRO A 2 43.400 18.130 45.770 1.00 0.17 C**

**ATOM 25 C PRO A 2 43.160 19.150 46.840 1.00 0.17 C**

**ATOM 26 O PRO A 2 43.820 19.110 47.840 1.00 0.17 O**

**ATOM 27 CB PRO A 2 42.990 16.730 46.190 1.00 0.17 C**

**ATOM 28 CG PRO A 2 43.980 15.930 46.350 1.00 0.17 C**

**ATOM 29 CD PRO A 2 45.210 16.560 45.830 1.00 0.17 C**

**ATOM 37 N ILE A 3 42.170 20.040 46.660 1.00 0.18 N**

**ATOM 38 CA ILE A 3 41.810 21.090 47.610 1.00 0.18 C**

**ATOM 39 C ILE A 3 40.320 20.960 47.890 1.00 0.18 C**

**ATOM 40 O ILE A 3 39.530 21.190 47.000 1.00 0.18 O**

**ATOM 41 CB ILE A 3 42.120 22.470 47.060 1.00 0.18 C**

**ATOM 42 CG1 ILE A 3 43.590 22.630 46.710 1.00 0.18 C**

**ATOM 43 CG2 ILE A 3 41.660 23.530 48.060 1.00 0.18 C**

**ATOM 44 CD ILE A 3 44.560 22.620 47.880 1.00 0.18 C**

**ATOM 56 N PRO A 4 39.850 20.660 49.090 1.00 0.21 N**

**ATOM 57 CA PRO A 4 38.430 20.690 49.400 1.00 0.21 C**

**ATOM 58 C PRO A 4 37.880 22.050 49.570 1.00 0.21 C**

**ATOM 59 O PRO A 4 38.420 22.790 50.280 1.00 0.21 O**

**ATOM 60 CB PRO A 4 38.390 19.900 50.660 1.00 0.21 C**

**ATOM 61 CG PRO A 4 39.650 19.780 51.180 1.00 0.21 C**

**ATOM 62 CD PRO A 4 40.630 20.120 50.120 1.00 0.21 C**

**ATOM 70 N LEU A 5 36.820 22.410 48.950 1.00 0.23 N**

**ATOM 71 CA LEU A 5 36.150 23.670 49.020 1.00 0.23 C**

**ATOM 72 C LEU A 5 34.690 23.430 49.390 1.00 0.23 C**

**ATOM 73 O LEU A 5 34.160 22.440 49.030 1.00 0.23 O**

**ATOM 74 CB LEU A 5 36.230 24.430 47.670 1.00 0.23 C**

**ATOM 75 CG LEU A 5 37.600 24.940 47.240 1.00 0.23 C**

**ATOM 76 CD1 LEU A 5 37.550 25.530 45.860 1.00 0.23 C**

**ATOM 77 CD2 LEU A 5 38.160 25.960 48.190 1.00 0.23 C**

**ATOM 89 N PRO A 6 34.030 24.270 50.130 1.00 0.23 N**

**ATOM 90 CA PRO A 6 32.590 24.280 50.340 1.00 0.23 C**

**ATOM 91 C PRO A 6 31.730 24.140 49.130 1.00 0.23 C**

**ATOM 92 O PRO A 6 32.170 24.500 48.090 1.00 0.23 O**

**ATOM 93 CB PRO A 6 32.320 25.570 51.050 1.00 0.23 C**

**ATOM 94 CG PRO A 6 33.520 25.910 51.560 1.00 0.23 C**

**ATOM 95 CD PRO A 6 34.640 25.290 50.860 1.00 0.23 C**

**ATOM 103 N TYR A 7 30.510 23.630 49.270 1.00 0.23 N**

**ATOM 104 CA TYR A 7 29.570 23.440 48.180 1.00 0.23 C**

**ATOM 105 C TYR A 7 29.290 24.680 47.340 1.00 0.23 C**

**ATOM 106 O TYR A 7 28.620 25.610 47.790 1.00 0.23 O**

**ATOM 107 CB TYR A 7 28.240 22.870 48.740 1.00 0.23 C**

**ATOM 108 CG TYR A 7 28.380 21.520 49.390 1.00 0.23 C**

**ATOM 109 CD1 TYR A 7 28.990 20.510 48.740 1.00 0.23 C**

**ATOM 110 CD2 TYR A 7 27.800 21.260 50.570 1.00 0.23 C**

**ATOM 111 CE1 TYR A 7 29.030 19.260 49.300 1.00 0.23 C**

**ATOM 112 CE2 TYR A 7 27.870 20.000 51.120 1.00 0.23 C**

**ATOM 113 CZ TYR A 7 28.500 19.000 50.470 1.00 0.23 C**

**ATOM 114 OH TYR A 7 28.540 17.750 51.010 1.00 0.23 O**

**ATOM 124 N TYR A 8 29.730 24.680 46.080 1.00 0.24 N**

**ATOM 125 CA TYR A 8 29.650 25.810 45.180 1.00 0.24 C**

**ATOM 126 C TYR A 8 28.240 26.230 44.830 1.00 0.24 C**

**ATOM 127 O TYR A 8 27.910 27.400 44.790 1.00 0.24 O**

**ATOM 128 CB TYR A 8 30.500 25.520 43.910 1.00 0.24 C**

**ATOM 129 CG TYR A 8 30.520 26.560 42.840 1.00 0.24 C**

**ATOM 130 CD1 TYR A 8 30.520 26.190 41.520 1.00 0.24 C**

**ATOM 131 CD2 TYR A 8 30.520 27.930 43.120 1.00 0.24 C**

**ATOM 132 CE1 TYR A 8 30.550 27.130 40.510 1.00 0.24 C**

**ATOM 133 CE2 TYR A 8 30.530 28.870 42.120 1.00 0.24 C**

**ATOM 134 CZ TYR A 8 30.550 28.480 40.790 1.00 0.24 C**

**ATOM 135 OH TYR A 8 30.580 29.420 39.800 1.00 0.24 O**

**ATOM 145 N TRP A 9 27.350 25.240 44.600 1.00 0.22 N**

**ATOM 146 CA TRP A 9 25.930 25.450 44.340 1.00 0.22 C**

**ATOM 147 C TRP A 9 25.170 26.120 45.450 1.00 0.22 C**

**ATOM 148 O TRP A 9 24.410 27.060 45.240 1.00 0.22 O**

**ATOM 149 CB TRP A 9 25.270 24.120 43.870 1.00 0.22 C**

**ATOM 150 CG TRP A 9 25.720 22.900 44.580 1.00 0.22 C**

**ATOM 151 CD1 TRP A 9 26.740 22.100 44.190 1.00 0.22 C**

**ATOM 152 CD2 TRP A 9 25.200 22.360 45.780 1.00 0.22 C**

**ATOM 153 CE2 TRP A 9 25.970 21.250 46.090 1.00 0.22 C**

**ATOM 154 CE3 TRP A 9 24.170 22.750 46.630 1.00 0.22 C**

**ATOM 155 NE1 TRP A 9 26.900 21.120 45.110 1.00 0.22 N**

**ATOM 156 CZ2 TRP A 9 25.730 20.500 47.200 1.00 0.22 C**

**ATOM 157 CZ3 TRP A 9 23.930 21.980 47.750 1.00 0.22 C**

**ATOM 158 CH2 TRP A 9 24.700 20.890 48.030 1.00 0.22 C**

**ATOM 169 N LEU A 10 25.440 25.720 46.690 1.00 0.21 N**

**ATOM 170 CA LEU A 10 24.920 26.360 47.870 1.00 0.21 C**

**ATOM 171 C LEU A 10 25.460 27.790 48.040 1.00 0.21 C**

**ATOM 172 O LEU A 10 24.740 28.740 48.270 1.00 0.21 O**

**ATOM 173 CB LEU A 10 25.200 25.490 49.110 1.00 0.21 C**

**ATOM 174 CG LEU A 10 24.770 25.990 50.490 1.00 0.21 C**

**ATOM 175 CD1 LEU A 10 23.320 26.290 50.560 1.00 0.21 C**

**ATOM 176 CD2 LEU A 10 25.180 25.030 51.570 1.00 0.21 C**

**ATOM 188 N TYR A 11 26.780 27.950 47.860 1.00 0.18 N**

**ATOM 189 CA TYR A 11 27.450 29.230 48.020 1.00 0.18 C**

**ATOM 190 C TYR A 11 26.990 30.290 47.040 1.00 0.18 C**

**ATOM 191 O TYR A 11 26.660 31.400 47.400 1.00 0.18 O**

**ATOM 192 CB TYR A 11 29.000 28.980 47.930 1.00 0.18 C**

**ATOM 193 CG TYR A 11 29.790 30.200 48.320 1.00 0.18 C**

**ATOM 194 CD1 TYR A 11 30.210 31.110 47.390 1.00 0.18 C**

**ATOM 195 CD2 TYR A 11 30.210 30.400 49.580 1.00 0.18 C**

**ATOM 196 CE1 TYR A 11 30.990 32.230 47.730 1.00 0.18 C**

**ATOM 197 CE2 TYR A 11 30.990 31.490 49.900 1.00 0.18 C**

**ATOM 198 CZ TYR A 11 31.380 32.400 48.980 1.00 0.18 C**

**ATOM 199 OH TYR A 11 32.190 33.460 49.290 1.00 0.18 O**

**ATOM 209 N ARG A 12 26.900 29.940 45.740 1.00 0.19 N**

**ATOM 210 CA ARG A 12 26.490 30.870 44.710 1.00 0.19 C**

**ATOM 211 C ARG A 12 25.040 31.280 44.830 1.00 0.19 C**

**ATOM 212 O ARG A 12 24.720 32.430 44.590 1.00 0.19 O**

**ATOM 213 CB ARG A 12 26.850 30.400 43.290 1.00 0.19 C**

**ATOM 214 CG ARG A 12 25.990 29.250 42.740 1.00 0.19 C**

**ATOM 215 CD ARG A 12 26.570 28.660 41.490 1.00 0.19 C**

**ATOM 216 NE ARG A 12 25.660 27.640 40.990 1.00 0.19 N**

**ATOM 217 CZ ARG A 12 25.960 26.420 40.690 1.00 0.19 C**

**ATOM 218 NH1 ARG A 12 27.080 25.920 40.850 1.00 0.19 N**

**ATOM 219 NH2 ARG A 12 25.080 25.660 40.200 1.00 0.19 N**

**ATOM 233 N ALA A 13 24.130 30.370 45.220 1.00 0.18 N**

**ATOM 234 CA ALA A 13 22.770 30.680 45.520 1.00 0.18 C**

**ATOM 235 C ALA A 13 22.630 31.640 46.690 1.00 0.18 C**

**ATOM 236 O ALA A 13 21.920 32.630 46.630 1.00 0.18 O**

**ATOM 237 CB ALA A 13 22.030 29.360 45.790 1.00 0.18 C**

**ATOM 243 N LEU A 14 23.390 31.410 47.790 1.00 0.18 N**

**ATOM 244 CA LEU A 14 23.400 32.330 48.910 1.00 0.18 C**

**ATOM 245 C LEU A 14 23.910 33.740 48.550 1.00 0.18 C**

**ATOM 246 O LEU A 14 23.280 34.730 48.850 1.00 0.18 O**

**ATOM 247 CB LEU A 14 24.200 31.680 50.030 1.00 0.18 C**

**ATOM 248 CG LEU A 14 24.270 32.370 51.330 1.00 0.18 C**

**ATOM 249 CD1 LEU A 14 23.440 32.290 52.090 1.00 0.18 C**

**ATOM 250 CD2 LEU A 14 25.150 32.210 52.110 1.00 0.18 C**

**ATOM 262 N ILE A 15 25.050 33.810 47.850 1.00 0.17 N**

**ATOM 263 CA ILE A 15 25.620 35.070 47.430 1.00 0.17 C**

**ATOM 264 C ILE A 15 24.760 35.810 46.420 1.00 0.17 C**

**ATOM 265 O ILE A 15 24.560 37.010 46.540 1.00 0.17 O**

**ATOM 266 CB ILE A 15 27.040 34.910 46.910 1.00 0.17 C**

**ATOM 267 CG1 ILE A 15 27.980 34.430 48.040 1.00 0.17 C**

**ATOM 268 CG2 ILE A 15 27.570 36.200 46.270 1.00 0.17 C**

**ATOM 269 CD ILE A 15 28.130 35.300 49.230 1.00 0.17 C**

**ATOM 281 N LYS A 16 24.170 35.120 45.450 1.00 0.16 N**

**ATOM 282 CA LYS A 16 23.190 35.740 44.560 1.00 0.16 C**

**ATOM 283 C LYS A 16 21.960 36.290 45.240 1.00 0.16 C**

**ATOM 284 O LYS A 16 21.500 37.370 44.880 1.00 0.16 O**

**ATOM 285 CB LYS A 16 22.810 34.820 43.390 1.00 0.16 C**

**ATOM 286 CG LYS A 16 23.940 34.610 42.360 1.00 0.16 C**

**ATOM 287 CD LYS A 16 24.420 35.850 41.640 1.00 0.16 C**

**ATOM 288 CE LYS A 16 23.430 36.530 40.780 1.00 0.16 C**

**ATOM 289 NZ LYS A 16 23.100 35.750 39.680 1.00 0.16 N**

**ATOM 303 N ARG A 17 21.440 35.630 46.270 1.00 0.13 N**

**ATOM 304 CA ARG A 17 20.390 36.200 47.130 1.00 0.13 C**

**ATOM 305 C ARG A 17 20.810 37.440 47.890 1.00 0.13 C**

**ATOM 306 O ARG A 17 20.060 38.410 47.960 1.00 0.13 O**

**ATOM 307 CB ARG A 17 19.780 35.130 48.050 1.00 0.13 C**

**ATOM 308 CG ARG A 17 18.960 34.010 47.370 1.00 0.13 C**

**ATOM 309 CD ARG A 17 17.740 34.480 46.620 1.00 0.13 C**

**ATOM 310 NE ARG A 17 18.110 34.800 45.240 1.00 0.13 N**

**ATOM 311 CZ ARG A 17 17.660 35.760 44.490 1.00 0.13 C**

**ATOM 312 NH1 ARG A 17 16.620 36.430 44.840 1.00 0.13 N**

**ATOM 313 NH2 ARG A 17 18.150 36.000 43.340 1.00 0.13 N**

**ATOM 327 N ILE A 18 22.030 37.470 48.430 1.00 0.12 N**

**ATOM 328 CA ILE A 18 22.590 38.650 49.070 1.00 0.12 C**

**ATOM 329 C ILE A 18 22.730 39.810 48.130 1.00 0.12 C**

**ATOM 330 O ILE A 18 22.370 40.940 48.440 1.00 0.12 O**

**ATOM 331 CB ILE A 18 23.870 38.340 49.800 1.00 0.12 C**

**ATOM 332 CG1 ILE A 18 23.660 37.400 50.970 1.00 0.12 C**

**ATOM 333 CG2 ILE A 18 24.590 39.590 50.250 1.00 0.12 C**

**ATOM 334 CD ILE A 18 23.930 37.450 51.800 1.00 0.12 C**

**ATOM 346 N GLN A 19 23.210 39.580 46.910 1.00 0.11 N**

**ATOM 347 CA GLN A 19 23.310 40.570 45.860 1.00 0.11 C**

**ATOM 348 C GLN A 19 21.970 41.070 45.340 1.00 0.11 C**

**ATOM 349 O GLN A 19 21.850 42.200 44.950 1.00 0.11 O**

**ATOM 350 CB GLN A 19 24.180 40.070 44.720 1.00 0.11 C**

**ATOM 351 CG GLN A 19 25.660 39.910 45.070 1.00 0.11 C**

**ATOM 352 CD GLN A 19 26.590 39.310 44.020 1.00 0.11 C**

**ATOM 353 NE2 GLN A 19 26.170 39.130 42.830 1.00 0.11 N**

**ATOM 354 OE1 GLN A 19 27.660 39.030 44.290 1.00 0.11 O**

**ATOM 363 N ALA A 20 20.960 40.210 45.340 1.00 0.10 N**

**ATOM 364 CA ALA A 20 19.600 40.620 45.050 1.00 0.10 C**

**ATOM 365 C ALA A 20 18.970 41.460 46.150 1.00 0.10 C**

**ATOM 366 O ALA A 20 18.150 42.310 45.860 1.00 0.10 O**

**ATOM 367 CB ALA A 20 18.770 39.370 44.850 1.00 0.10 C**

**ATOM 373 N LEU A 21 19.330 41.250 47.410 1.00 0.09 N**

**ATOM 374 CA LEU A 21 18.900 42.060 48.540 1.00 0.09 C**

**ATOM 375 C LEU A 21 19.440 43.460 48.490 1.00 0.09 C**

**ATOM 376 O LEU A 21 18.760 44.420 48.790 1.00 0.09 O**

**ATOM 377 CB LEU A 21 19.280 41.370 49.850 1.00 0.09 C**

**ATOM 378 CG LEU A 21 18.890 42.070 51.140 1.00 0.09 C**

**ATOM 379 CD1 LEU A 21 17.460 42.110 51.350 1.00 0.09 C**

**ATOM 380 CD2 LEU A 21 19.480 41.410 52.260 1.00 0.09 C**

**ATOM 392 N ILE A 22 20.700 43.630 48.120 1.00 0.09 N**

**ATOM 393 CA ILE A 22 21.370 44.900 48.080 1.00 0.09 C**

**ATOM 394 C ILE A 22 21.160 45.530 46.750 1.00 0.09 C**

**ATOM 395 O ILE A 22 21.490 44.920 45.790 1.00 0.09 O**

**ATOM 396 CB ILE A 22 22.800 44.750 48.390 1.00 0.09 C**

**ATOM 397 CG1 ILE A 22 23.050 44.130 49.710 1.00 0.09 C**

**ATOM 398 CG2 ILE A 22 23.460 46.080 48.300 1.00 0.09 C**

**ATOM 399 CD ILE A 22 23.880 43.900 50.250 1.00 0.09 C**

**ATOM 411 N PRO A 23 20.610 46.710 46.560 1.00 0.09 N**

**ATOM 412 CA PRO A 23 20.220 47.190 45.270 1.00 0.09 C**

**ATOM 413 C PRO A 23 21.370 47.630 44.450 1.00 0.09 C**

**ATOM 414 O PRO A 23 21.390 47.370 43.320 1.00 0.09 O**

**ATOM 415 CB PRO A 23 19.290 48.320 45.520 1.00 0.09 C**

**ATOM 416 CG PRO A 23 19.300 48.470 46.830 1.00 0.09 C**

**ATOM 417 CD PRO A 23 19.910 47.430 47.560 1.00 0.09 C**

**ATOM 425 N LYS A 24 22.310 48.300 44.980 1.00 0.09 N**

**ATOM 426 CA LYS A 24 23.480 48.690 44.310 1.00 0.09 C**

**ATOM 427 C LYS A 24 24.630 48.480 45.220 1.00 0.09 C**

**ATOM 428 O LYS A 24 24.510 48.680 46.390 1.00 0.09 O**

**ATOM 429 CB LYS A 24 23.440 50.100 43.830 1.00 0.09 C**

**ATOM 430 CG LYS A 24 22.470 50.480 42.810 1.00 0.09 C**

**ATOM 431 CD LYS A 24 22.560 50.180 41.610 1.00 0.09 C**

**ATOM 432 CE LYS A 24 22.150 50.490 40.760 1.00 0.09 C**

**ATOM 433 NZ LYS A 24 21.990 50.820 39.970 1.00 0.09 N**

**ATOM 447 N GLY A 25 25.770 48.060 44.730 1.00 0.09 N**

**ATOM 448 CA GLY A 25 26.910 47.720 45.500 1.00 0.09 C**

**ATOM 449 C GLY A 25 26.770 46.440 46.170 1.00 0.09 C**

**ATOM 450 O GLY A 25 25.850 45.730 45.990 1.00 0.09 O**

**ATOM 454 N GLY A 26 27.740 46.070 46.940 1.00 0.11 N**

**ATOM 455 CA GLY A 26 27.750 44.930 47.780 1.00 0.11 C**

**ATOM 456 C GLY A 26 27.940 43.660 47.090 1.00 0.11 C**

**ATOM 457 O GLY A 26 27.380 42.680 47.450 1.00 0.11 O**

**ATOM 461 N ARG A 27 28.700 43.640 46.030 1.00 0.14 N**

**ATOM 462 CA ARG A 27 28.970 42.470 45.300 1.00 0.14 C**

**ATOM 463 C ARG A 27 30.150 41.710 45.840 1.00 0.14 C**

**ATOM 464 O ARG A 27 31.200 42.190 45.950 1.00 0.14 O**

**ATOM 465 CB ARG A 27 29.110 42.780 43.820 1.00 0.14 C**

**ATOM 466 CG ARG A 27 27.830 43.030 43.080 1.00 0.14 C**

**ATOM 467 CD ARG A 27 26.830 43.840 43.560 1.00 0.14 C**

**ATOM 468 NE ARG A 27 25.630 43.830 42.790 1.00 0.14 N**

**ATOM 469 CZ ARG A 27 24.430 44.030 43.190 1.00 0.14 C**

**ATOM 470 NH1 ARG A 27 24.120 44.490 44.300 1.00 0.14 N**

**ATOM 471 NH2 ARG A 27 23.450 43.740 42.420 1.00 0.14 N**

**ATOM 485 N LEU A 28 29.980 40.470 46.230 1.00 0.16 N**

**ATOM 486 CA LEU A 28 30.900 39.640 46.950 1.00 0.16 C**

**ATOM 487 C LEU A 28 31.530 38.590 46.060 1.00 0.16 C**

**ATOM 488 O LEU A 28 32.370 37.900 46.490 1.00 0.16 O**

**ATOM 489 CB LEU A 28 30.170 38.920 48.100 1.00 0.16 C**

**ATOM 490 CG LEU A 28 29.540 39.790 49.130 1.00 0.16 C**

**ATOM 491 CD1 LEU A 28 28.720 39.360 49.980 1.00 0.16 C**

**ATOM 492 CD2 LEU A 28 30.300 40.360 49.810 1.00 0.16 C**

**ATOM 504 N LEU A 29 31.120 38.490 44.820 1.00 0.17 N**

**ATOM 505 CA LEU A 29 31.730 37.670 43.830 1.00 0.17 C**

**ATOM 506 C LEU A 29 31.580 36.170 44.030 1.00 0.17 C**

**ATOM 507 O LEU A 29 32.420 35.570 44.670 1.00 0.17 O**

**ATOM 508 CB LEU A 29 33.170 38.080 43.520 1.00 0.17 C**

**ATOM 509 CG LEU A 29 33.480 39.290 42.950 1.00 0.17 C**

**ATOM 510 CD1 LEU A 29 34.480 39.810 42.930 1.00 0.17 C**

**ATOM 511 CD2 LEU A 29 33.340 39.480 41.920 1.00 0.17 C**

**ATOM 523 N PRO A 30 30.560 35.490 43.520 1.00 0.20 N**

**ATOM 524 CA PRO A 30 30.260 34.140 43.930 1.00 0.20 C**

**ATOM 525 C PRO A 30 31.270 33.100 43.550 1.00 0.20 C**

**ATOM 526 O PRO A 30 31.350 32.100 44.200 1.00 0.20 O**

**ATOM 527 CB PRO A 30 28.920 33.820 43.290 1.00 0.20 C**

**ATOM 528 CG PRO A 30 28.510 34.980 42.530 1.00 0.20 C**

**ATOM 529 CD PRO A 30 29.480 36.070 42.740 1.00 0.20 C**

**ATOM 537 N GLN A 31 32.010 33.320 42.470 1.00 0.23 N**

**ATOM 538 CA GLN A 31 32.940 32.360 41.920 1.00 0.23 C**

**ATOM 539 C GLN A 31 34.360 32.570 42.360 1.00 0.23 C**

**ATOM 540 O GLN A 31 35.200 31.750 42.030 1.00 0.23 O**

**ATOM 541 CB GLN A 31 32.860 32.360 40.370 1.00 0.23 C**

**ATOM 542 CG GLN A 31 33.270 33.620 39.650 1.00 0.23 C**

**ATOM 543 CD GLN A 31 32.650 34.830 39.910 1.00 0.23 C**

**ATOM 544 NE2 GLN A 31 32.370 35.220 39.380 1.00 0.23 N**

**ATOM 545 OE1 GLN A 31 32.430 35.410 40.560 1.00 0.23 O**

**ATOM 554 N LEU A 32 34.600 33.610 43.140 1.00 0.24 N**

**ATOM 555 CA LEU A 32 35.920 34.070 43.520 1.00 0.24 C**

**ATOM 556 C LEU A 32 36.930 33.060 44.050 1.00 0.24 C**

**ATOM 557 O LEU A 32 37.970 32.850 43.470 1.00 0.24 O**

**ATOM 558 CB LEU A 32 35.770 35.220 44.520 1.00 0.24 C**

**ATOM 559 CG LEU A 32 37.020 35.890 45.080 1.00 0.24 C**

**ATOM 560 CD1 LEU A 32 37.870 36.510 44.010 1.00 0.24 C**

**ATOM 561 CD2 LEU A 32 36.660 36.940 46.080 1.00 0.24 C**

**ATOM 573 N VAL A 33 36.600 32.340 45.140 1.00 0.23 N**

**ATOM 574 CA VAL A 33 37.530 31.360 45.680 1.00 0.23 C**

**ATOM 575 C VAL A 33 37.760 30.170 44.760 1.00 0.23 C**

**ATOM 576 O VAL A 33 38.850 29.660 44.650 1.00 0.23 O**

**ATOM 577 CB VAL A 33 37.200 30.870 47.060 1.00 0.23 C**

**ATOM 578 CG1 VAL A 33 37.350 31.680 47.910 1.00 0.23 C**

**ATOM 579 CG2 VAL A 33 36.120 30.410 47.370 1.00 0.23 C**

**ATOM 589 N TYR A 34 36.700 29.730 44.060 1.00 0.21 N**

**ATOM 590 CA TYR A 34 36.750 28.660 43.090 1.00 0.21 C**

**ATOM 591 C TYR A 34 37.610 29.040 41.910 1.00 0.21 C**

**ATOM 592 O TYR A 34 38.370 28.240 41.420 1.00 0.21 O**

**ATOM 593 CB TYR A 34 35.320 28.250 42.620 1.00 0.21 C**

**ATOM 594 CG TYR A 34 34.490 27.950 43.830 1.00 0.21 C**

**ATOM 595 CD1 TYR A 34 34.480 26.680 44.390 1.00 0.21 C**

**ATOM 596 CD2 TYR A 34 33.740 28.950 44.430 1.00 0.21 C**

**ATOM 597 CE1 TYR A 34 33.730 26.400 45.520 1.00 0.21 C**

**ATOM 598 CE2 TYR A 34 33.000 28.710 45.590 1.00 0.21 C**

**ATOM 599 CZ TYR A 34 33.000 27.430 46.130 1.00 0.21 C**

**ATOM 600 OH TYR A 34 32.220 27.160 47.240 1.00 0.21 O**

**ATOM 610 N ARG A 35 37.570 30.310 41.480 1.00 0.18 N**

**ATOM 611 CA ARG A 35 38.460 30.840 40.480 1.00 0.18 C**

**ATOM 612 C ARG A 35 39.900 30.970 40.970 1.00 0.18 C**

**ATOM 613 O ARG A 35 40.820 30.650 40.260 1.00 0.18 O**

**ATOM 614 CB ARG A 35 37.950 32.180 39.910 1.00 0.18 C**

**ATOM 615 CG ARG A 35 36.800 32.030 38.910 1.00 0.18 C**

**ATOM 616 CD ARG A 35 37.170 31.580 37.550 1.00 0.18 C**

**ATOM 617 NE ARG A 35 37.260 30.200 37.510 1.00 0.18 N**

**ATOM 618 CZ ARG A 35 38.040 29.540 36.800 1.00 0.18 C**

**ATOM 619 NH1 ARG A 35 38.770 30.040 35.960 1.00 0.18 N**

**ATOM 620 NH2 ARG A 35 38.070 28.350 36.910 1.00 0.18 N**

**ATOM 634 N LEU A 36 40.180 31.400 42.220 1.00 0.17 N**

**ATOM 635 CA LEU A 36 41.530 31.520 42.760 1.00 0.17 C**

**ATOM 636 C LEU A 36 42.220 30.180 42.890 1.00 0.17 C**

**ATOM 637 O LEU A 36 43.400 30.070 42.680 1.00 0.17 O**

**ATOM 638 CB LEU A 36 41.460 32.220 44.130 1.00 0.17 C**

**ATOM 639 CG LEU A 36 41.290 33.560 44.220 1.00 0.17 C**

**ATOM 640 CD1 LEU A 36 41.200 34.130 45.430 1.00 0.17 C**

**ATOM 641 CD2 LEU A 36 42.120 34.350 43.720 1.00 0.17 C**

**ATOM 653 N VAL A 37 41.480 29.130 43.250 1.00 0.17 N**

**ATOM 654 CA VAL A 37 41.980 27.800 43.410 1.00 0.17 C**

**ATOM 655 C VAL A 37 41.930 27.010 42.110 1.00 0.17 C**

**ATOM 656 O VAL A 37 42.460 25.960 41.980 1.00 0.17 O**

**ATOM 657 CB VAL A 37 41.230 27.120 44.540 1.00 0.17 C**

**ATOM 658 CG1 VAL A 37 41.670 25.710 44.790 1.00 0.17 C**

**ATOM 659 CG2 VAL A 37 41.360 27.950 45.840 1.00 0.17 C**

**ATOM 669 N LEU A 38 41.320 27.550 41.080 1.00 0.16 N**

**ATOM 670 CA LEU A 38 41.110 26.940 39.780 1.00 0.16 C**

**ATOM 671 C LEU A 38 40.290 25.690 39.800 1.00 0.16 C**

**ATOM 672 O LEU A 38 40.690 24.650 39.380 1.00 0.16 O**

**ATOM 673 CB LEU A 38 42.390 26.780 38.960 1.00 0.16 C**

**ATOM 674 CG LEU A 38 43.140 28.040 38.690 1.00 0.16 C**

**ATOM 675 CD1 LEU A 38 44.460 27.750 38.030 1.00 0.16 C**

**ATOM 676 CD2 LEU A 38 42.400 28.960 37.820 1.00 0.16 C**

**ATOM 688 N ARG A 39 39.090 25.730 40.340 1.00 0.15 N**

**ATOM 689 CA ARG A 39 38.120 24.670 40.380 1.00 0.15 C**

**ATOM 690 C ARG A 39 36.840 25.100 39.690 1.00 0.15 C**

**ATOM 691 O ARG A 39 36.550 26.300 39.660 1.00 0.15 O**

**ATOM 692 CB ARG A 39 37.800 24.190 41.800 1.00 0.15 C**

**ATOM 693 CG ARG A 39 38.990 23.580 42.530 1.00 0.15 C**

**ATOM 694 CD ARG A 39 39.600 22.380 41.890 1.00 0.15 C**

**ATOM 695 NE ARG A 39 40.660 21.800 42.720 1.00 0.15 N**

**ATOM 696 CZ ARG A 39 41.950 21.900 42.540 1.00 0.15 C**

**ATOM 697 NH1 ARG A 39 42.500 22.820 41.840 1.00 0.15 N**

**ATOM 698 NH2 ARG A 39 42.720 21.040 43.070 1.00 0.15 N**

**ATOM 712 N TYR A 40 36.080 24.160 39.110 1.00 0.15 N**

**ATOM 713 CA TYR A 40 34.860 24.400 38.380 1.00 0.15 C**

**ATOM 714 C TYR A 40 35.020 25.180 37.090 1.00 0.15 C**

**ATOM 715 O TYR A 40 35.060 26.200 36.980 1.00 0.15 O**

**ATOM 716 CB TYR A 40 33.690 24.960 39.240 1.00 0.15 C**

**ATOM 717 CG TYR A 40 33.210 23.930 40.210 1.00 0.15 C**

**ATOM 718 CD1 TYR A 40 33.600 23.890 41.510 1.00 0.15 C**

**ATOM 719 CD2 TYR A 40 32.290 23.060 39.830 1.00 0.15 C**

**ATOM 720 CE1 TYR A 40 33.080 23.030 42.420 1.00 0.15 C**

**ATOM 721 CE2 TYR A 40 31.790 22.160 40.730 1.00 0.15 C**

**ATOM 722 CZ TYR A 40 32.180 22.140 42.010 1.00 0.15 C**

**ATOM 723 OH TYR A 40 31.650 21.260 42.910 1.00 0.15 O**

**ATOM 733 N SER A 41 35.070 24.690 36.060 1.00 0.16 N**

**ATOM 734 CA SER A 41 35.180 25.310 34.780 1.00 0.16 C**

**ATOM 735 C SER A 41 33.970 25.280 34.010 1.00 0.16 C**

**ATOM 736 CB SER A 41 36.200 24.730 33.980 1.00 0.16 C**

**ATOM 737 OG SER A 41 36.430 25.350 32.770 1.00 0.16 O**

**ATOM 738 OT1 SER A 41 33.410 24.920 33.700 1.00 0.16 O**

**ATOM 739 OT2 SER A 41 33.540 25.610 33.760 1.00 0.16 O**

**TER 740 SER A 41**

**END**
